# Education of healthcare professionals to improve guideline adherence in atrial fibrillation: the STEEER-AF cluster-randomized clinical trial

**DOI:** 10.1038/s41591-025-03751-2

**Published:** 2025-06-13

**Authors:** Dipak Kotecha, Karina V. Bunting, Samir Mehta, Philipp Sommer, Maciej Sterliński, Kim Rajappan, Lluís Mont, Eduard Guasch, Serge Boveda, Giuseppe Boriani, Yongzhong Sun, Colinda van Deutekom, Chris P. Gale, Tom J. R. De Potter, Isabelle C. van Gelder, on behalf of the STEEER-AF investigators, Yann Allali, Asgher Champsi, Thomas Deneke, Kaitlyn Greeley, Benoît Guy-Moyat, Mikael Laredo, Alastair Mobley, Maximina Ventura, Mary Stanbury, Trudie Lobban, Thompson Robinson, Tatjana Potpara, Eloi Marijon, Pascal Defaye, Pierre Baudinaud, Simon Kochhaeuser, Ursula Rauch, Moritz F. Sinner, Marco Proietti, Igor Diemberger, Vincenzo Russo, Stanislaw Tubek, Piotr Buchta, Pawel Balsam, Eusebio García-Izquierdo, Ivo Roca Luque, Jose M. Guerra, Dewi Thomas, Afzal Sohaib, Mark J. Davies, Olivier Piot, William Escande, Christian De Chillou, Maxime De Guillebon, Frédéric Anselme, Andrea Cianci, Rodrigue Garcia, Philippe Maury, Dominique Pavin, Estelle Gandjbakhch, Frédéric Sacher, Karim Hasni, Fabien Garnier, Charles Guenancia, Nicolas Lellouche, Stephan Willems, Martin Borlich, Andreas Metzner, Hans-Holger Ebert, Dong-In Shin, David Duncker, Stefan G. Spitzer, Peter Nordbeck, Roland R. Tilz, Andrea Mazza, Cinzia Valzania, Margherita Padeletti, Matteo Bertini, Jacopo F. Imberti, Stefano Fumagalli, Antonio Rapacciuolo, Monika Lica Gorzynska, Adam Gorlo, Marcin Kostkiewicz, Grzegorz Sobieszek, Andrzej S. Skrzyński, Robert Gajda, Hanna Wilk-Manowiec, Jaroslaw Blicharz, Wiktor K. Gmiński, Tomasz Czerski, Felipe Bisbal, Ignasi Anguera, Teresa Lozano, Joaquin Osca, Jose L. Merino, Naiara Calvo, Juan Fernández-Armenta, Juan Acosta, Nuria Rivas-Gandara, Pilar Cabanas-Grandío, Emilce Trucco, Richard Bond, Richard Ang, Shawn A. A. Morais, Fu Siong Ng, Matthew G. D. Bates, Michala Pedersen, Daniel T. Raine, Manish Kalla, Matthew J. Lovell, Malcolm Finlay, Arif Hasan Bhuiyan, Norman Qureshi, Hein Heidbuchel, Wolfram Döhner, Bernard Iung, Susanna Price, Helmut Pürerfellner, Barbara Casadei, Alex R. Lyon, Winston Banya, Robert Hatala, Pekka Raatikainen, Paulus Kirchhof

**Affiliations:** 1 Department of Cardiovascular Sciences, University of Birmingham, Birmingham, UK; 2 Cardiology Department, University Hospitals Birmingham National Health Service Foundation Trust, Birmingham, UK; 3 NIHR Birmingham Biomedical Research Centre, Birmingham, UK; 4 Birmingham Clinical Trials Unit, Institute of Applied Health Research, Birmingham, UK; 5 Clinic for Electrophysiology, Heart and Diabetes Center North Rhine-Westphalia, Bad Oeynhausen, Germany; 6 Arrhythmia Center, National Institute of Cardiology, Warsaw, Poland; 7 Cardiology Department, Oxford University Hospitals National Health Service Foundation Trust, Oxford, UK; 8 Cardiovascular Institute, Hospital Clínic, Universitat de Barcelona, Barcelona, Spain; 9 Institut d’Investigacions Biomèdiques August Pi i Sunyer, Barcelona, Spain; 10 Centro de Investigación Médica en Red—Enfermedades Cardiovasculares, Madrid, Spain; 11 Heart Rhythm Management Department, Cardiology, Clinique Pasteur, Toulouse, France; 12 Vrije Universiteit Brussels, Brussels, Belgium; 13 Division of Cardiology, Department of Biomedical, Metabolic and Neural Sciences, Policlinico di Modena, University of Modena and Reggio Emilia, Modena, Italy; 14 Department of Cardiology, University of Groningen, University Medical Centre Groningen, Groningen, the Netherlands; 15 Leeds Institute for Cardiovascular and Metabolic Medicine, University of Leeds, Leeds, UK; 16 Leeds Institute of Data Analytics, University of Leeds, Leeds, UK; 17 Department of Cardiology, Leeds Teaching Hospitals, Leeds, UK; 18 Cardiovascular Center, Aalst, Belgium; 19 Cardiology Institute, Electrophysiology Unit, Pitié Salpêtrière University Hospital, APHP, Paris, France; 20 Clinic for Arrhythmology, Klinikum Nuernberg, University Hospital of the Paracelsus Medical University, Nuernberg, Germany; 21 Cardiology Division, European Georges Pompidou hospital, Paris, France; 22 CHU De Limoges Dupuytren 2, Limoges, France; 23 Patient and public involvement team, University of Birmingham, Birmingham, UK; 24 Patient and public involvement team, Arrhythmia Alliance, Chipping Norton, UK; 25 College of Life Sciences and National Institute for Health and Care Research Biomedical Research Centre, University of Leicester, Leicester, UK; 26 Cardiology Clinic, Clinical Centre of Serbia, Belgrade, Serbia; 27 University Hospital Grenoble Alpes, Grenoble, France; 28 Department of Arrhythmia Services, Marienhospital, Osnabrück, Germany; 29 Department of Cardiology, Angiology and Intensive Care, Charité—Universitätsmedizin Berlin, Berlin, Germany; 30 Medical Department I, University Hospital Munich, Munich, Germany; 31 Department of Clinical Sciences and Community Health Maugeri, University of Milan, Milan, Italy; 32 Institute of Cardiology, Department of Medical and Surgical Sciences, Policlinico S.Orsola-Malpighi, University of Bologna, Bologna, Italy; 33 Department of Translational Medical Sciences, University of Campania Luigi Vanvitelli, Naples, Italy; 34 Institute of heart diseases, University Hospital Wroclaw, Wroclaw, Poland; 35 3rd Department of Cardiology, Faculty of Medical Sciences in Zabrze, Medical University of Silesia, Katowice, Poland; 36 1St Department of Cardiology, Medical University Of Warsaw, Warsaw, Poland; 37 Arrhythmia Unit, Hospital Universitario Puerta De Hierro, Madrid, Spain; 38 Hospital de la Santa Creu i Sant Pau, Barcelona, Spain; 39 Morriston Hospital Regional Cardiac Centre, Swansea Bay University Health Board, Swansea, UK; 40 Department of Cardiology, St Bartholomew’s Hospital, Barts Health National Health Service Trust, London, UK; 41 Oxford University Hospitals NHS Foundation Trust; Milton Keynes University Hospital National Health Service Foundation Trust, Oxford, UK; 42 Electrophysiology, Centre Cardiologique Du Nord, Saint-Denis, France; 43 Cardiology Unit, Institut Lorrain du Cœur et des Vaisseaux, Centre Hospitalier Régional Universitaire de Nancy, Vandoeuvre lès Nancy, France; 44 Cardiology Unit, Centre Hospitalier de Pau, Pau, France; 45 CHU de Rouen, Rouen, France; 46 Cardiology, Electrophysiology Unit, University Hospital of Poitiers, Poitiers, France; 47 Department of Cardiology, University Hospital Rangueil Toulouse, Toulouse, France; 48 Cardiology, Centre Hospitalier Universitaire de Rennes, Rennes, France; 49 Centre de Recherche Cardio-Thoracique de Bordeaux, Université de Bordeaux, Bordeaux, France; 50 Cardiology Unit, Electrophysiology Unit, Sainte Musse Hospital, Toulon, France; 51 Cardiology Unit, Centre Hospitalier Universitaire de Dijon Bourgogne, Dijon, France; 52 Cardiology Unit, Electrophysiology Unit, Hopital Henri Mondor APHP, Créteil, France; 53 Cardiology and internal intensive care medicine, Asklepios Clinic St. Georg, Hamburg, Germany; 54 Cardiology Heart and Vascular Center, Segeberger Kliniken, Bad Segeberg, Germany; 55 University Heart and Vascular Center Hamburg, Hamburg, Germany; 56 Cardiology Unit, Devicetherapy, Kardiologische Gemeinschaftspraxis Riesa, Riesa, Germany; 57 Faculty of Health, School of Medicine, University Witten/Herdecke, Witten, Germany; 58 Hannover Heart Rhythm Center, Department of Cardiology and Angiology, Hannover Medical School, Hannover, Germany; 59 Heart and Vessel Clinic, Dresden, Germany; 60 University Hospital Wuerzburg, Würzburg, Germany; 61 University Heart Center Lübeck, Department of Rhythmology, University Hospital Schleswig-Holstein, Lübeck, Germany; 62 Emergency Department, Cardiology, Orvieto Hospital, Orvieto, Italy; 63 Cardiology and Electrophysiology Unit, Santa Maria Nuova Hospital, Florence, Italy; 64 University Hospital Sant’Anna, Ferrara, Italy; 65 Department of Experimental and Clinical Medicine, University of Florence, Geriatric Intensive Care Unit, Florence, Italy; 66 Advanced Biomedical Sciences, Cardiology, Federico II University, Naples, Italy; 67 Cardiology, District Hospital, Chojnice, Poland; 68 Electrophysiology, Cardiology, Cardiovita, Suwałki, Poland; 69 Cardiology Department, Medical Care Center, Jarosław, Poland; 70 Cardiology Clinic, Cardiology, 1 Military Hospital, Lublin, Poland; 71 Internal Unit, Independent Public Healthcare Centre Lukow, Łuków, Poland; 72 Gajda-Med District Hospital in Pultusk, Pułtusk, Poland; 73 Cardiology, Siedlce 2 Hospital, Siedlce, Poland; 74 Cardiology, Szpital Wojewodzki in Sw. Lukasza - Regional Specialist Hospital, Tarnów, Poland; 75 Voivodeship Hospital Combined, Cardiology Department, Toruń, Poland; 76 Internal Medicine and Cardiology, Hospital Wegrow, Węgrów, Poland; 77 Heart Institute (iCor), Electrophysiology Dept, Hospital Germans Trias i Pujol, Badalona, Spain; 78 Cardiology, Arryhtmia Unit, Bellvitge University Hospital, L’Hospitalet, Spain; 79 Department of Cardiology, Hospital General Universitario Doctor Balmis. Instituto de Investigación Sanitaria y Biomédica de Alicante, Alicante, Spain; 80 Arrhythmia Section, Cardiology Department, University and Polytechnic Hospital la Fe, Valencia, Spain; 81 Arrhythmia and Robotic EP Unit, La Paz University Hospital, Madrid, Spain; 82 Cardiology, Arryhtmia Unit, Hospital Universitario Miguel Servet, Zaragoza, Spain; 83 Cardiology, Arryhtmia Unit, Hospital Universitario Puerta del Mar, Cádiz, Spain; 84 Cardiology, Arryhtmia Unit, Virgen del Rocío University Hospital, Seville, Spain; 85 Cardiology, Arryhtmia Unit, Vall d’Hebron Hospital, Barcelona, Spain; 86 Cardiology, Arryhtmia Unit, Hospital Álvaro Cunqueiro, Vigo, Spain; 87 Arrhythmia Section, Cardiology Department, Hospital Universitari Doctor Josep Trueta and Institut d’Investigació Biomèdica de Girona, Girona, Spain; 88 Cardiology, Medicine, Gloucestershire Royal Hospitals National Health Service Foundation Trust, Gloucester, UK; 89 Cardiology Department, Homerton Healthcare National Health Service Foundation Trust, London, UK; 90 Cardiology, Medicine, Rehabilitation and Cardiac, Oxford University Hospital Trust, Banbury, UK; 91 National Heart and Lung Institute, Faculty of Medicine, Imperial College London, London, UK; 92 Cardiology Unit, The James Cook University Hospital, Middlesbrough, UK; 93 Department of Cardiology, Northampton General Hospital National Health Service Trust, Northampton, UK; 94 Cardiac Electrophysiology, Norfolk and Norwich University Hospital, Norwich, UK; 95 Department of Cardiology, Queen Elizabeth Hospital, Birmingham, UK; 96 Cardiology, Medicine, Royal Devon and Exeter Hospital, Exeter, UK; 97 Cardiac Electrophysiology, Barts Heart Centre, Barts Health National Health Service Trust, London, UK; 98 Cardiology, Medicine, Barts Health National Health Service Trust, London, UK; 99 Cardiology, Buckinghamshire Healthcare National Health Service Trust, London, UK; 100 Department of Cardiology, Antwerp University Hospital, Antwerp, Belgium; 101 Berlin Institute of Health, Center for Regenerative Therapies, Charité—Universitätsmedizin Berlin, Berlin, Germany; 102 Cardiology Unit, Bichat Hospital, Assistance Publique—Hôpitaux de Paris, Paris, France; 103 Royal Brompton Hospital, London, UK; 104 Ordensklinikum Linz Elisabethinen, Cardiology, Linz, Austria; 105 National Institute for Health and Care Research Oxford Biomedical Research Centre, Univeristy of Oxford, Oxford, UK; 106 Arrhythmias and Pacing, Cardiology and Angiology, National Institute of Cardiovascular Diseases, Bratislava, Slovakia; 107 Cardiology, Heart and Lung Center Helsinki University Hospital, Helsinki, Finland

**Keywords:** Atrial fibrillation, Health services, Randomized controlled trials

## Abstract

Guideline-adherent care is associated with better patient outcomes, but whether this can be achieved by professional education is unclear. Here we conducted a cluster-randomized controlled trial across 70 centers in six countries to understand if a program for the education of healthcare professionals could improve patient-level adherence to clinical practice guidelines on atrial fibrillation (AF). Each center recruited patients with AF seen in routine practice (total *N* = 1,732), after which the centers were randomized, accounting for baseline guideline adherence to class I and III recommendations from the European Society of Cardiology on stroke prevention and rhythm control. Healthcare professionals in the intervention centers received a 16-week structured educational program with an average of 9 h of online engagement, whereas those at control centers received no additional education beyond standard practice. For the co-primary stroke prevention outcome, guideline adherence was 63.4% and 58.6% at baseline and 67.5% and 60.9% at 6–9-months follow-up for the intervention and control groups, respectively (adjusted risk ratio 1.10; 95% confidence interval (CI) 0.97 to 1.24; *P* = 0.13). For the co-primary rhythm control outcome, guideline adherence was 21.4% and 20.4% at baseline and 33.9% and 22.9% at follow-up for the intervention and control groups, respectively (adjusted risk ratio 1.51; 95% CI 1.04 to 2.18; *P* = 0.03). The secondary outcome of patient-reported integrated AF management showed a 5.1% improvement in the intervention group compared with the control group (95% CI 1.4% to 8.9%; *P* = 0.01). Thus, while the education of healthcare professionals improved substantial gaps in implementation for rhythm control, it had no significant effect on stroke prevention. ClinicalTrials.gov registration: NCT04396418.

## Abstract (web summary)

In a cluster-randomized trial conducted across 70 medical centers in six countries, a 16-week structured educational program for healthcare providers improved adherence to guidelines for rhythm control but not for stroke prevention in individuals with atrial fibrillation.

## Main

Considerable effort and resources are expended globally to educate and train healthcare professionals to improve outcomes for patients^1^. The effectiveness of these programs is highly variable and dependent on technique, interactivity and subject context^2,3^. Clinical practice guidelines are widely used around the world to standardize care and provide optimal patient management according to the current evidence base. However, implementation of guideline-adherent care is often challenging, especially in conditions with high patient heterogeneity where delivery of the optimal treatment pathway is dependent on individual clinical factors. One such condition is atrial fibrillation (AF), which is already one of the most common cardiovascular conditions and expected to double in prevalence in the coming decades^4,5^. There is a substantial risk of morbidity and mortality associated with AF^6,7^, but this is highly variable and requires an individualized approach and often difficult decision-making on best-practice management^8^. Guideline-adherent care for anticoagulation therapy in patients with AF^9,10^ has been associated with lower rates of stroke, bleeding and death^11–13^.

The extent of guideline implementation is challenging to ascertain in observational research although typically thought to be poor, with numerous barriers implicated such as appropriate education^14–16^. Patient education has the potential to reduce serious adverse events and have a positive impact on quality of life in patients with AF^17^. However, educational interventions directed at healthcare professionals to improve the adherence to cardiovascular guidelines have had limited success^18^. The Stroke prevention and rhythm control Therapy Evaluation of an Educational Program of the European society of cardiology in a Cluster Randomized trial in patients with Atrial Fibrillation (STEEER-AF) trial was designed to robustly determine real-world adherence to clinical practice guidelines and examine the value of an educational intervention directed to a range of healthcare professionals treating patients with AF^19,20^. The primary objective was to establish if the addition of concise structured learning for healthcare professionals could improve patient-level adherence to guidelines for stroke prevention and rhythm control compared with standard practice.

## Results

**Fig. 1:**
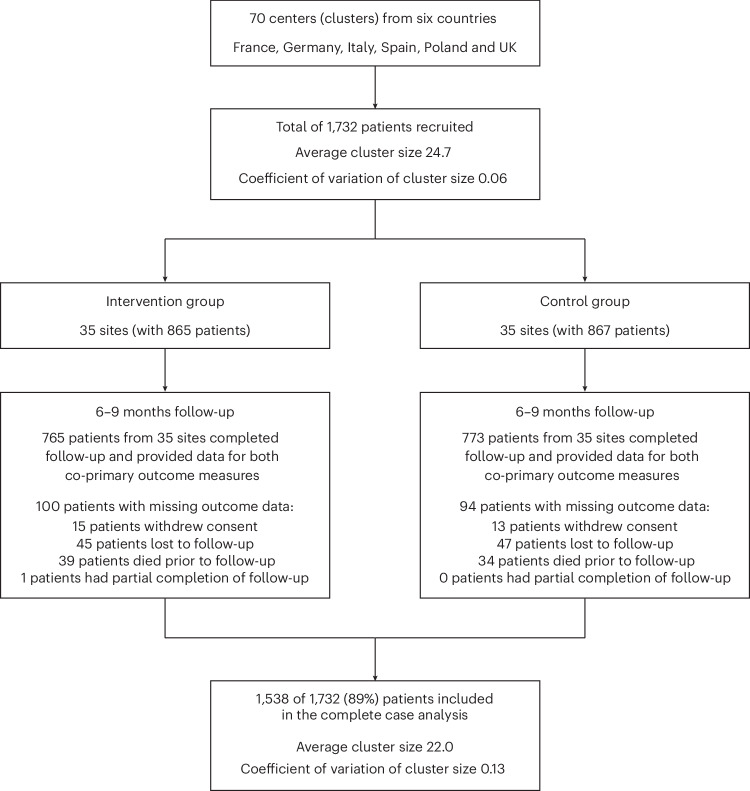
STEEER-AF trial flowchart. CONSORT diagram for the centers and patients enrolled in the trial. Each center was randomized only after patient recruitment had been completed, aiming for a maximum of 25 patients per center. A 1:1 randomization to intervention (additional education for healthcare professionals) or control (usual approaches to medical education) was performed using a minimization algorithm to account for country and the cluster-level values for the co-primary outcomes at baseline. All centers randomized to the intervention group received the educational program.

Seventy centers across France, Germany, Italy, Poland, Spain and the UK were included, with center characteristics summarized in Extended Data Table 1. A total of 1,732 patients with AF were recruited, with average cluster size of 24.7 patients (coefficient of variation in cluster size 0.06) (Fig. 1). The mean age of participants was 68.9 years (s.d. 11.7), with 647 (37.4%) women and similar baseline characteristics between the randomized groups (Table 1). The randomization of centers took place only after participant recruitment had closed (between May 2022 and February 2023), with 35 centers allocated to the intervention and 35 to the control. The minimization algorithm ensured that randomization was balanced within each country for baseline guideline adherence (Extended Data Table 2).

**Table 1:** Baseline characteristics

Characteristic	Intervention (*N* = 865)	Control (*N* = 867)	Total (*N* = 1,732)
Age at enrollment, mean (s.d.), years	68.7 (11.6)	69.0 (11.7)	68.9 (11.7)
Women, *n* (%)	340 (39.3%)	307 (35.4%)	647 (37.4%)
Type of AF			
First diagnosed	147 (17.0%)	135 (15.6%)	282 (16.3%)
Paroxysmal	340 (39.3%)	316 (36.4%)	656 (37.9%)
Persistent	228 (26.4%)	256 (29.5%)	484 (27.9%)
Long-standing persistent	38 (4.4%)	28 (3.2%)	66 (3.8%)
Permanent	112 (12.9%)	132 (15.2%)	244 (14.1%)
Duration of AF			
≤1 year	346 (40.0%)	336 (38.8%)	682 (39.4%)
1–5 years	256 (29.6%)	286 (33.0%)	542 (31.3%)
>5 years	263 (30.4%)	245 (28.3%)	508 (29.3%)
Rhythm on electrocardiogram^a^			
Sinus rhythm	401 (46.4%)	359 (41.4%)	760 (43.9%)
AF	407 (47.1%)	436 (50.3%)	843 (48.7%)
Diagnosis of COVID-19	150 (17.3%)	122 (14.1%)	272 (15.7%)
Hypertension requiring treatment	588 (68.0%)	619 (71.4%)	1,207 (69.7%)
Diabetes mellitus	181 (20.9%)	205 (23.6%)	386 (22.3%)
History of stroke or TIA	74 (8.6%)	102 (11.8%)	176 (10.2%)
History of myocardial infarction	86 (9.9%)	80 (9.2%)	166 (9.6%)
Diagnosis of heart failure	272 (31.4%)	217 (25.0%)	489 (28.2%)
Left-ventricular ejection fraction			
Preserved (≥50%)	645 (74.6%)	643 (74.2%)	1,288 (74.4%)
Mildly reduced (40–49%)	104 (12.0%)	103 (11.9%)	207 (12.0%)
Reduced (<40%)	116 (13.4%)	121 (14.0%)	237 (13.7%)
Resting heart rate on ECG, beats min^−1^ (s.d.)^b^	78.8 (22.9)	79.7 (23.4)	79.2 (23.2)
Systolic blood pressure, mmHg (s.d.)^b^	131.7 (18.7)	132.0 (20.7)	131.8 (19.7)
Diastolic blood pressure, mmHg (s.d.)^b^	77.5 (11.8)	78.2 (13.1)	77.8 (12.5)
Body mass index, kg/m^2^ (s.d.)^b^	28.9 (6.1)	28.3 (5.5)	28.6 (5.8)
Creatinine, μmol/L (s.d.); mg dL^−1^ (s.d.)^b^	121.7 (285.3); 1.38 (3.23)	124.0 (190.2); 1.40 (2.15)	122.8 (243.4); 1.39 (2.75)
Taking an oral anticoagulant at baseline	779 (90.1%)	764 (88.1%)	1543 (89.1%)
Receiving antiarrhythmic drugs at baseline	266 (30.8%)	285 (32.9%)	551 (31.8%)
Catheter ablation performed before baseline	201 (23.2%)	142 (16.4%)	343 (19.8%)

COVID-19, coronavirus disease 2019; TIA, transient ischemic attack.

^a^129 patients had another rhythm or pacing at baseline.

^b^Missing data: 20 patients for ECG heart rate, 52 for systolic blood pressure, 53 for diastolic blood pressure, 38 for body mass index and 205 for creatinine.

### Baseline adherence to guidelines

Guideline adherence was evaluated for each individual patient using the pre-published decision tree algorithm^20^. The observed guideline adherence at baseline to all relevant class I and III European Society of Cardiology (ESC) recommendations for stroke prevention overall was 61.0% (intracluster correlation coefficient 0.11). In the intervention group, 548 patients (63.4%) were fully adherent for stroke prevention, with 508 (58.6%) in the control group. For rhythm control, overall guideline adherence at baseline was 21.0% (intracluster correlation coefficient 0.26); there were 185 patients (21.4%) in the intervention group fully adherent and 178 (20.5%) in the control group.

### Co-primary outcomes

**Fig. 2:**
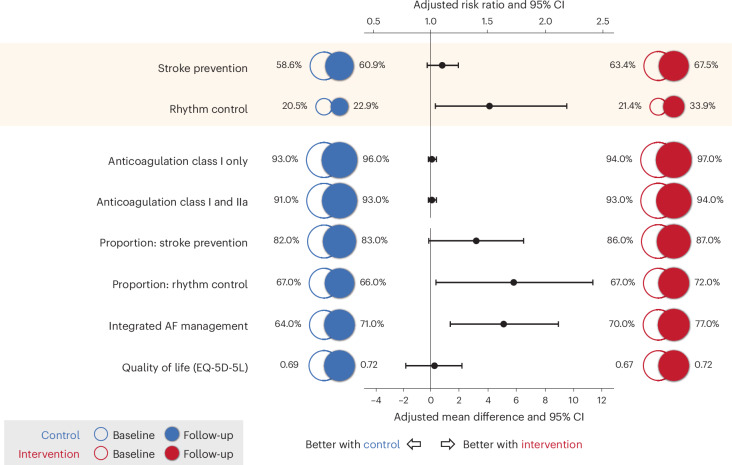
Co-primary and secondary outcomes. Outcomes are presented as a risk ratio or adjusted mean difference for intervention versus control, with circles for point estimates and capped lines for the 95% CI adjusted for baseline values, country and clustering by center. For each outcome, absolute values are indicated at baseline and follow-up for each of the groups. The shaded area indicates the co-primary trial outcomes.

The median time between randomization and completion of the follow-up electronic case report forms (eCRF) was 7.7 months (interquartile range (IQR) 5.9–8.8 months). For stroke prevention at follow-up, guideline adherence increased to 516 patients (67.5%) in the intervention group and 471 (60.9%) for control (Table 2 and Fig. 2). The risk ratio for intervention versus control after adjusting for baseline values, country and clustering by center was 1.10 (95% confidence interval (CI) 0.97 to 1.24; *P* = 0.13). The corresponding adjusted risk difference was 6.0% (95% CI −1.5% to 13.4%; *P* = 0.12). For rhythm control at follow-up, guideline adherence increased to 259 patients (33.9%) in the intervention group and 177 (22.9%) for control. The adjusted risk ratio for intervention versus control was significant at 1.51 (95% CI 1.04 to 2.18; *P* = 0.03), with adjusted risk difference 11.2% (95% CI 1.6 to 20.7; *P* = 0.02).

**Table 2:** Primary and secondary outcomes

Outcome	Intervention		Control		Risk ratio (95% CI); *P* value	Risk or mean difference (95% CI); *P* value
	Baseline (*N* = 865)	Follow-up (*N* = 765)	Baseline (*N* = 867)	Follow-up (*N* = 773)		
**Co-primary outcomes**						
Stroke prevention guideline adherence, *n* (%)	548 (63.4%)	516 (67.5%)	508 (58.6%)	471 (60.9%)	1.10 (0.97 to 1.24), *P* = 0.13	6.0% (−1.5% to 13.4%), *P* = 0.12
Rhythm control guideline adherence, *n* (%)	185 (21.4%)	259 (33.9%)	178 (20.5%)	177 (22.9%)	1.51 (1.04 to 2.18), *P* = 0.03	11.2% (1.6% to 20.7%), *P* = 0.02
**Secondary outcomes**						
Anticoagulation class I indication, *n* (%)^a^	608 (94.4%)	559 (97.2%)	602 (92.8%)	556 (95.7%)	1.02 (0.99 to 1.05), *P* = 0.26	1.6% (−1.1% to 4.3%), *P* = 0.26
Anticoagulation class I and IIa, *n* (%)^b^	723 (93.1%)	649 (94.5%)	717 (91.2%)	653 (93.3%)	1.01 (0.98 to 1.05), *P* = 0.40	1.3% (−1.7% to 4.3%), *P* = 0.40
Proportion of relevant guidelines with adherence for stroke prevention, mean (s.d.)	0.86 (0.21)	0.87 (0.20)	0.82 (0.25)	0.83 (0.25)	–	3.2% (−0.1% to 6.5%), *P* = 0.06
Proportion of relevant guidelines with adherence for rhythm control, mean (s.d.)	0.67 (0.27)	0.72 (0.28)	0.67 (0.25)	0.66 (0.27)	–	5.8% (0.4% to 11.3%), *P* = 0.04
Patient-reported integrated AF management, mean (s.d.)	0.70 (0.28)	0.77 (0.26)	0.64 (0.29)	0.71 (0.26)	–	5.1% (1.4% to 8.9%), *P* = 0.01
Patient-reported quality of life						
EQ-5D-5L index score, mean (s.d.)	0.84 (0.20)	0.81 (0.28)	0.83 (0.21)	0.80 (0.27)	–	0.00 (−0.03% to 0.03%), *P* = 0.95
Visual analog scale, mean (s.d.)	67.3 (19.3)	71.6 (18.5)	68.6 (19.1)	71.6 (16.7)	–	0.3 (−1.7% to 2.2%), *P* = 0.79

All risk ratios, risk differences and mean differences are for intervention versus control at follow-up, adjusted for baseline values, minimization criteria (country, baseline guideline adherence for stroke prevention and rhythm control) and clustering by center. Risk differences and differences in proportions are presented in percentage points.

^a^The proportion of participants receiving anticoagulation according to the class I ESC recommendation (women with CHA_2_DS_2_-VASc score ≥3 and men ≥2); intervention *N* = 644 at baseline and *N* = 575 at follow-up control and *N* = 649 at baseline and *N* = 581 at follow-up.

^b^The proportion of participants receiving anticoagulation according to Class I or IIa ESC recommendations (women with CHA_2_DS_2_-VASc score ≥2 and men ≥1) intervention; *N* = 777 at baseline and *N* = 687 at follow-up and control *N* = 786 at baseline and *N* = 700 at follow-up.

### Secondary outcomes

Prescriptions of oral anticoagulation according to class I and class I/IIa indications were high (94% and 92% at baseline) and not impacted by the intervention (risk ratio 1.02, 95% CI 0.99 to 1.05; *P* = 0.26 and 1.01, 95% CI 0.98 to 1.05; *P* = 0.40, respectively; Table 2 and Fig. 2). The proportions of class I and III stroke prevention and rhythm control guidelines with adherence were consistent with the co-primary outcomes (adjusted mean difference in proportions of 3.2% for stroke prevention, 95% CI −0.1% to 6.5%; *P* = 0.06 and 5.8% for rhythm control, 95% CI 0.4% to 11.3%; *P* = 0.04). The proportion of applicable patients failing at each point in the guideline decision tree was variable, with key factors for both stroke prevention (Extended Data Table 3) and rhythm control (Extended Data Table 4) being appropriate evaluation and patient indication(s) for the therapeutic approach. Patients in the intervention group reported a significant 5.1% improvement over control in the proportion attaining integrated AF management (95% CI 1.4% to 8.9%; *P* = 0.01). There were no differences between groups in patient-reported quality of life.

### Process outcomes

Learners spent a median of 9.2 h (IQR 6.4–13.4 h) on the online platform (Extended Data Table 5), with a high level of involvement with required reading (mean 94.8%, s.d. 9.7) and with their commitment to change local practice (97.8% implemented). The expert trainer was engaged by 158 of 195 learners (81.0%). The proportion of correct answers to multiple-choice questions increased with the intervention, from a mean of 65.2% before the education (s.d. 18.3%) to 72.0% after (s.d. 19.9%) (post-hoc *P* < 0.001).

### Subgroup and sensitivity analyses

**Fig. 3:**
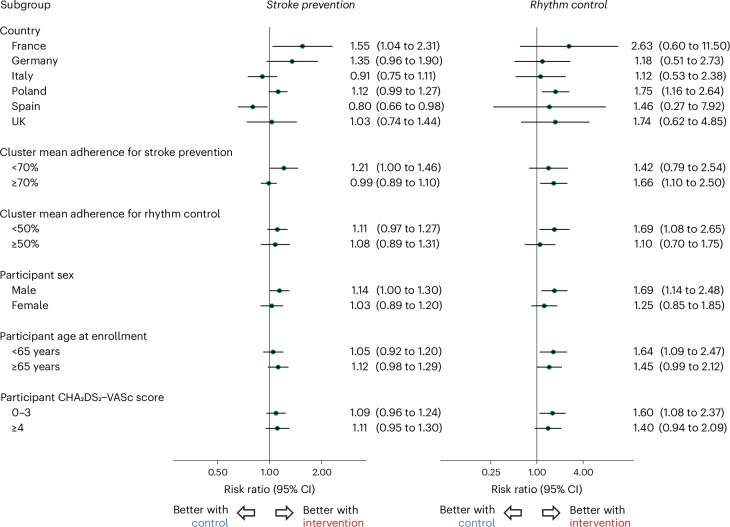
Subgroup analyses for co-primary outcomes. Prespecified subgroup analyses are presented for the two co-primary outcomes, with circles for point estimates of risk ratios and lines for the 95% CI.

**Extended Data Fig. 1:**
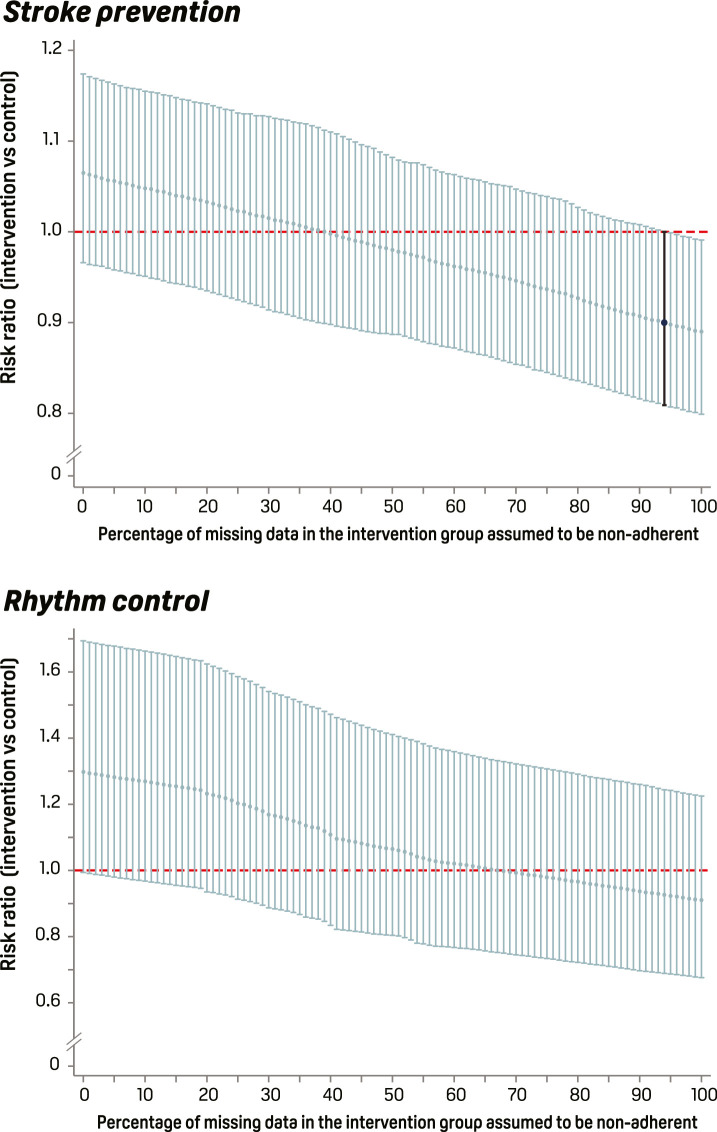
Tipping Point Sensitivity Analyses. Graphs show the tipping point at which results will favor the control arm if participants with missing data at follow-up are imputed as being non-adherent to guidelines in the intervention arm and adherent in the control arm. Panel A for the stroke prevention co-primary outcome demonstrates that the tipping point only occurs after 95% of the missing data is imputed as non-adherent, and all of the missing data in the control arm are imputed as adherent. Panel B for the rhythm control co-primary outcome demonstrates that even if all missing data is imputed as non-adherent in the intervention arm and adherent in the control arm, this will still not provide a result that statistically favors the control group.

Exploratory analyses indicated consistent effects for the co-primary outcomes across subgroups for patient age and baseline thromboembolic risk (Fig. 3). Effects were noted to vary by country for stroke prevention (*P* interaction = 0.01; Extended Data Table 6) and sex for rhythm control (*P*_interaction_ = 0.01; Extended Data Table 7). The sensitivity analysis confirmed robust findings for both co-primary outcomes (Extended Data Fig. 1).

## Discussion

STEEER-AF demonstrates that adherence to the ‘must-do’ and ‘must-do not’ guideline recommendations in routine practice is poor, in particular the implementation of rhythm control in patients with AF. The educational intervention for healthcare professionals in the STEEER-AF trial resulted in a 51% relative increase (11.2% absolute increase) in patient-level adherence to recommendations for rhythm management. This benefit resulted from a relatively short and targeted educational program in addition to, and compared against, existing approaches for continued medical education. We saw no significant difference for guideline adherence to recommendations on the prevention of stroke and thromboembolism in AF.

Findings for the secondary outcomes were consistent with the co-primary results and help explain the divergence in results of STEEER-AF across the two components of AF care. The prescription of oral anticoagulants, which form the basis of stroke prevention in the majority of patients, was high. A key patient-reported secondary outcome demonstrated the intervention improved integration of care, with education, shared decision-making and the empowerment of patients being important facets of delivering optimal and individualized rhythm control.

Overall guideline adherence in the STEEER-AF trial was substantially lower than anticipated from prior observational research, where self-reporting can introduce response bias^21^. Our study suggests the need for a reappraisal of strategies to improve the delivery of patient care and enhance guideline adoption by guideline writers, professional associations, medical educators and policy makers. Prior evidence for improving guideline-adherent care is largely restricted to the use of oral anticoagulation in patients with AF, including a cluster trial which demonstrated higher rates of anticoagulant prescription with education compared with usual care (odds ratio 3.28, 95% CI 1.67 to 6.44), and lower rates of the secondary outcome of incident stoke in the intervention group (hazard ratio 0.48, 95% CI 0.23 to 0.99)^22^. Of note, that trial was performed in Argentina, Brazil, China, India and Romania, with 68% of patients prescribed an oral anticoagulant at baseline. On the basis of the findings from STEEER-AF and these other studies, implementing approaches to guideline-adherent management is likely to require focused education that is tailored not only to a specific delivery gap, but also the needs of the individual healthcare professional in the context of the local healthcare environment. Although we observed an improvement in rhythm control adherence, additional approaches are clearly needed to tailor education for patients and healthcare professionals, further improving guideline adherence and patient outcomes. A trial of shared decision-making did not identify any benefit for anticoagulation use in AF or related safety endpoints^23^, although it could be argued that shared care is dependent on adequate levels of education for both patients and healthcare staff.

This trial was designed to test the value of education specifically for healthcare professionals, as prior trials have already demonstrated that patient education, with the right approach and format, can yield benefits for patients with AF^17,24^. We accounted for a range of inherent biases that are common when assessing the value of an educational intervention. Recruitment of participants at each site was completed before randomization could take place, and extraction of the clinical pathway for each patient was determined objectively rather than from the healthcare professional involved. Any imbalance in randomization was minimized using the cluster-level adherence of the co-primary outcomes calculated at baseline, and the algorithm to determine guideline adherence was pre-defined but not disclosed. Although we were unable to blind healthcare professionals to the randomized allocation at their center, all coordinating staff were kept blinded. Care was taken to avoid any contamination of trial groups by geographically dispersing sites across each country. The enrolled participants were a good reflection of patients with AF seen in real-world cohort studies^25–27^. Despite considerable external events (such as the coronavirus pandemic, which precluded in-person training of healthcare professionals^20^), there was good engagement with the bespoke online educational platform that was specifically developed to achieve sustainable behavioral change, supported by national trained experts.

The findings of STEEER-AF on targeted education for healthcare professionals, combined with past research on targeted education of patients, would suggest an important opportunity to achieve clinical and societal benefit through scalable multifactorial interventions. These approaches need to be part of broader quality improvement efforts that are ongoing in local environments, national policy-making and also on the international level (for example, World Health Organization Quality of Care in line with the United Nations Sustainable Development Goals; the European Commission’s European Education Area initiative; and various strategies on education and quality improvement by the Association of American Medical Colleges).

There are limitations that warrant consideration. First, the trial was established to ascertain the value of additional education; hence both intervention and control groups could engage in whatever usual approaches were available to meet their educational needs. As a pragmatic trial embedded within clinical practice, it was not possible to determine or account for varying levels of education within or across the six countries^20^. However, all the countries involved are members of the ESC and European Heart Rhythm Association (EHRA), which have the same core curriculum for cardiology that includes AF^28^. Second, the co-primary outcomes were focused on determining adherence to class I and III recommendations (that is, where there is no dispute that treatments are either effective or harmful). This leaves out many areas of routine practice that are important to patient care. Although the ESC guidelines have contribution from the national cardiac societies in each country, there can be different applications of best practice locally. The STEEER-AF was deployed across 70 centers in six countries so that regional or national differences in implementation were accounted for by design. Third, the trial was conducted in the European secondary care setting, and the findings may not apply to primary care, lower-income areas or environments with a different approach to professional development. Although care was taken to engage a broad range of centers within each country, these sites did agree to participate, which may indicate an existing interest in quality improvement. The trial was powered to detect clinically relevant changes in guideline adherence within the first year, rather than clinical outcomes, which will be explored in future reports. Finally, the sample size assumptions deviated from actuality (lower baseline adherence using our objective trial assessment than expected from available observational data), and so the trial may be underpowered to detect the anticipated differences in stroke prevention management.

In summary, the STEEER-AF trial showed that guideline adherence in patients with AF is poor. Focused education for healthcare professionals did not demonstrate positive effects on guideline-adherent care for both co-primary outcomes. Improvement was noted in rhythm control where guideline implementation was particularly low, but with no significant effect on recommendations addressing stroke prevention where anticoagulation use was near-optimal.

## Methods

### Trial design and oversight

STEEER-AF is an international, pragmatic, two parallel group, cluster-randomized controlled trial, supported in its design by a patient and public involvement team^19,20^. The full protocol is available in the additional files (no changes after trial commencement). A cluster design was the most suitable approach for testing the educational program for healthcare professionals, as effect contamination could occur with individual patient-level randomization. The trial was sponsored by the ESC, with contribution from the EHRA and ESC Council on Stroke. A trial management group and trial steering committee directed the program, with oversight provided by an independent data monitoring committee and strategic oversight committee (Extended Data Table 8).

### Ethics and inclusion statement

The trial was approved by ethical review committees in each country and local research governance authorities for each center. A patient and public involvement team aided with the design of the concept and drafting of patient-facing material to improve inclusivity. The trial was prospectively registered at clinicaltrials.gov (NCT04396418).

### Participating centers, investigators and patients

The cluster-level selection criteria were a site that agreed to participation, enrollment and follow-up of patients and randomization of the site to the intervention or control for healthcare practitioners at that center. A national coordinator for each country was tasked with engaging a broad range of centers in their country that treat patients with AF and were representative of usual care, and selected a local principal investigator (PI) for each site. Healthcare professionals were nominated by the PI from across different specialties within each center to act as investigators, including trainee and experienced doctors, nurses and allied health professionals, with no more than a third of investigators seeing patients with AF on a daily basis. Each investigator recruited patients who were under their routine clinical care, with a maximum of 25 patients per center. Participants required a clinical diagnosis of AF and the ability to provide written informed consent. Patient-level exclusion criteria were age under 18 years, pregnancy or planning to be pregnant, breastfeeding at the time of consent, participation in another clinical trial of an investigational medicinal product or device and life expectancy of less than 2 years. Participants were followed up in routine practice by the same investigator at 6–9 months after each center was randomized. Where that was not possible (for example, the healthcare professional had left that institution), follow-up was performed by another investigator from that center.

### Randomization and masking

Centers were randomized only after they had finished participant recruitment and fully completed baseline eCRF, managed by an independent contract research organization (Soladis). To provide objective assessment of the clinical care received, the eCRF was completed by the PI who was not involved in the care pathway for recruited participants. The eCRF was completed after the interaction between patient and investigator using all available clinical and/or electronic documentation. The PI received queries for any missing elements on the eCRF forms. Algorithms were used to objectively determine guideline adherence at the level of each patient. These algorithms were finalized and approved before the first randomization and are available in an open-access publication (10.1093/europace/euae178)^20^.

The algorithms were applied to the eCRF data for each participant. Guideline adherence was not disclosed to the PI or investigators to avoid influencing follow-up. Randomization was performed by the Birmingham Clinical Trials Unit (University of Birmingham), with a 1:1 ratio to intervention or control using a minimization algorithm to ensure balance by (1) country, (2) cluster-specific mean for class I and III guideline adherence to stroke prevention at baseline (<70 and ≥70%) and (3) cluster-specific mean for class I and III guideline adherence to rhythm control at baseline (<50 and ≥50%). The randomized allocation was performed by the trial statistician blinded to the identity of the centers. Owing to the nature of the intervention, it was not possible to blind investigators to the randomized allocation. The trial steering committee were blinded to the randomized allocation of centers during the entire trial.

### Intervention and control

The educational intervention for healthcare professionals was targeted toward stroke prevention, rhythm control and integrated care in AF, with learning modules translated to the language for each participating country. Investigators from centers randomized to the intervention group were enrolled in an additional educational program lasting 16 weeks, primarily consisting of online resources. The intervention was designed by the ESC and EHRA, taking advantage of decades of work in methods applied to better educate healthcare professionals^1^, and utilizing educational theory and learning frameworks to achieve sustainable behavioral change^29^. The educational intervention was developed with the assistance of an independent medical education agency (Liberum IME), with further details published previously^20^.

The web-based platform included course materials, interaction with peers, case-based learning, videos and additional reading (providing direct educational benefit). Learners were supported by an expert trainer from that country that assisted with case-based examples of appropriate guideline-adherent care, and helped them to generate a ‘commitment to change’ plan that could be implemented locally to improve the management of patients with AF (indirect benefits from the educational program).

Centers randomized to the control group did not receive the additional educational intervention, but investigators were able to continue any existing healthcare professional development.

### Outcomes

Full details on the outcomes are presented in the protocol. The co-primary outcomes were guideline adherence for stroke prevention and rhythm control on the basis of class I and III ESC recommendations from the 2016 and 2020 guidelines on the management of AF^9,10^. The prespecified secondary outcomes were the proportion of guidelines with adherence for stroke prevention and rhythm control, and the proportion of participants receiving anticoagulation according to class I and class I/IIa indications. The key patient-reported outcome was a score evaluating eight domains of integrated AF management, completed by the patient after their consultation with the investigator. Patient-reported quality of life was determined using the EuroQol EQ-5D-5L questionnaire (index values and visual analog scale). Process outcomes in the intervention group addressed the fidelity of the educational program. Ongoing follow-up is in process to collect future clinical outcomes.

### Sample size

Sample size calculations for the stroke prevention co-primary outcome assumed that 80% of control patients expected to receive guideline-adherent care based on available observational studies^30,31^. A relative increase of 10% was considered clinically relevant (absolute increase from 80% to 88%). For this co-primary outcome, power was 85% based on an intracluster correlation coefficient of 0.04 (refs. ^22,32^), two-sided alpha 0.05, cluster size of 25 patients, coefficient of variation in cluster size 0.20, 70 clusters and 10% loss of patients to follow-up. For the rhythm control co-primary outcome, estimates of guideline-adherent care for rhythm control in the control group were 50% (refs. ^30,33^). Using the same assumptions as the stroke prevention co-primary outcome, the power to detect an absolute increase from 50% to 61% was 85%.

### Statistical analysis

A statistical analysis plan was finalized and approved before unlocking the trial database (see additional file). All analyses were performed according to the intention-to-treat principle (according to the randomized allocation). The primary comparison was between the centers (clusters) randomized to the intervention group and those randomized to the control group. All model-based analyses were adjusted for minimization criteria (country and baseline guideline adherence for stroke prevention and rhythm control), the baseline of that variable (where appropriate) and clustering for center. All analyses were performed on patient-level data and used patient-level covariate adjustments. For each of the co-primary outcomes, we fitted a generalized linear mixed model using the binomial distribution and logit link (with robust standard error), followed by marginal standardization to estimate the risk ratio and risk difference. Type I error control is not required for co-primary endpoints^34^. Statistical analyses for the co-primary outcomes were double-coded by an independent statistician in a separate statistical package (Stata; StataCorp) to the analyses conducted by the senior statistician (SAS; SAS Institute). Prespecified subgroup analyses for the co-primary outcomes were the minimization variables and participant age, sex and CHA_2_DS_2_-VASc score at baseline (with 2 points for age ≥75 years and prior stroke, transient ischemic attack or systematic embolus, and 1 point for chronic heart failure, hypertension, diabetes mellitus, vascular disease, age ≥65 years or female sex). An additional planned subgroup analysis according to the modified EHRA symptom classification score was not pursued owing to missing data for this variable. Effects within these subgroups were examined by including the relevant subgroup by intervention interaction term. To examine the possible impact of any missing data on the co-primary outcome results, a prespecified sensitivity analysis explored whether missing outcomes were ‘missing not at random’ using a tipping point approach. Secondary outcomes with binary data were analyzed using the same methods as described for the co-primary outcomes, and differences in proportions were analyzed using a fractional regression model with logit link and cluster-robust standard errors. Secondary outcomes with continuous data were analyzed using mixed effects linear regression to estimate the adjusted mean difference.

### Role of the funding source

The ESC and EHRA (not-for-profit professional organizations) contributed to the study design and data collection. The external funders provided educational grants to the ESC and had no role in study design, data collection, data analysis, data interpretation or writing of the report.

### Reporting frameworks

The study is reported according to the cluster randomized trial extension of the CONSORT checklist (see additional file).

### Reporting summary

Further information on research design is available in the Nature Portfolio Reporting Summary linked to this article.

## Online content

Any methods, additional references, Nature Portfolio reporting summaries, source data, extended data, supplementary information, acknowledgments, peer review information; details of author contributions and competing interests; and statements of data and code availability are available at 10.1038/s41591-025-03751-2.

## Supplementary information

Supplementary Information CONSORT cluster checklist, trial protocol and statistical analysis plan.

Reporting Summary

## Figures and Tables

**Extended Data Table 1:**
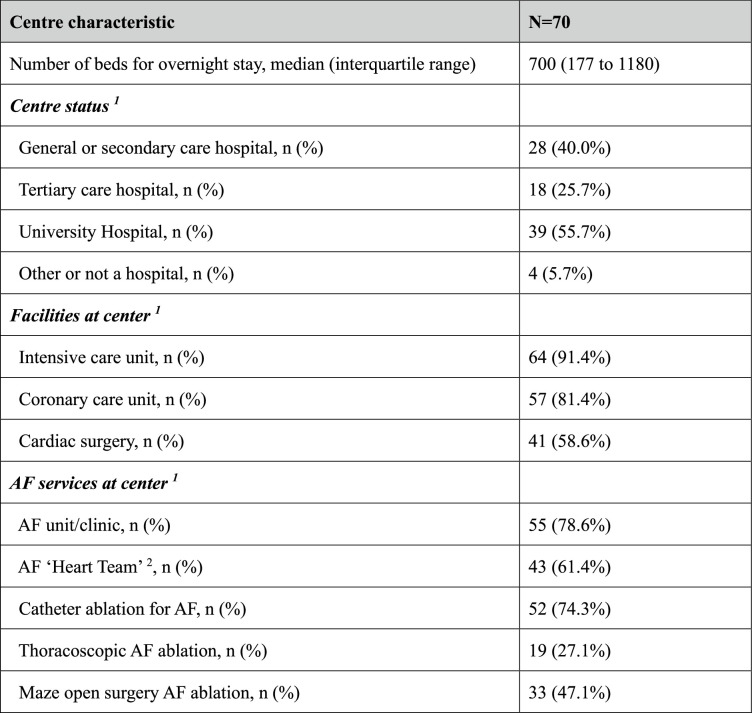
Characteristics of Included Centers

^1^Not mutually exclusive; multiple possible for each center.

^2^Multidisciplinary complex case review by cardiologists, electrophysiologists, AF surgeons or other relevant specialists. AF = atrial fibrillation.

**Extended Data Table 2:**
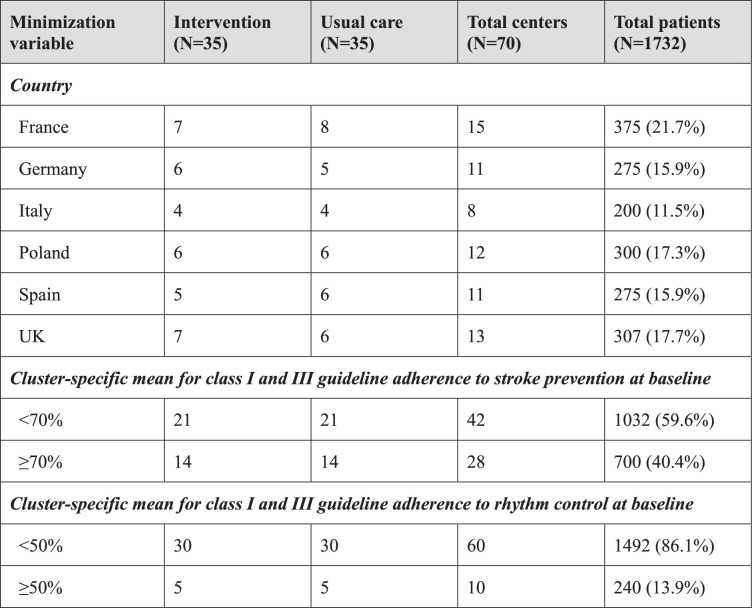
Randomization of Centers

**Extended Data Table 3:**
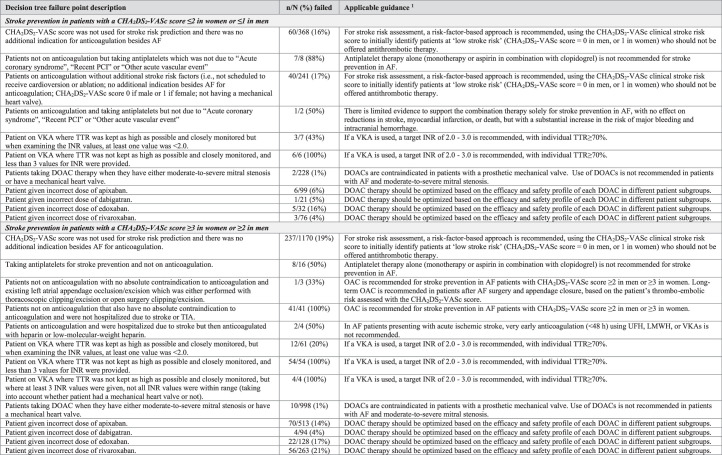
Guideline Non-Adherence For Stroke Prevention

Table presents data for failure points with any number of applicable patients, as determined using the pre-published guideline decision tree algorithm [10.1093/europace/euae178]^20^.

^1^From the European Society of Cardiology guidelines for the management of atrial fibrillation [10.1093/eurheartj/ehw210 and 10.1093/eurheartj/ehaa612]^9,10^. AF = atrial fibrillation; CHA_2_DS_2_-VASc = stroke/thromboembolism risk score [Congestive heart failure, Hypertension, Age ≥75 years (2 points), Diabetes mellitus, Stroke or previous thromboembolism (2 points), Vascular disease, Age 65–74 years, Sex category (female)]; INR = International normalized ratio; LMWH = low molecular weight heparin; DOAC = direct oral anticoagulant; OAC = oral anticoagulation; PCI = percutaneous coronary intervention; TIA = transient ischemic attack; TTR = time in therapeutic range; UFH = unfractionated heparin; VKA = vitamin K antagonist oral anticoagulant.

**Extended Data Table 4:**
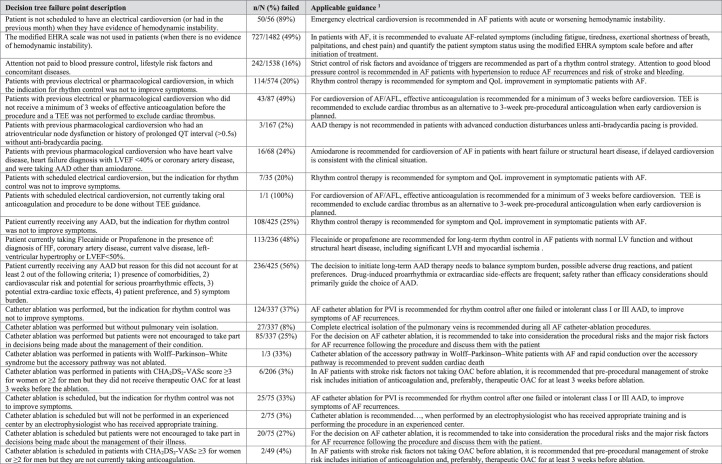
Guideline Non-Adherence for Rhythm Control

Table presents data for failure points with any number of applicable patients, as determined using the pre-published guideline decision tree algorithm [10.1093/europace/euae178]^20^.

^1^From the European Society of Cardiology guidelines for the management of atrial fibrillation [10.1093/eurheartj/ehw210 and 10.1093/eurheartj/ehaa612]^9,10^. AF = atrial fibrillation; CHA_2_DS_2_-VASc = stroke/thromboembolism risk score [Congestive heart failure, Hypertension, Age ≥75 years (2 points), Diabetes mellitus, Stroke or previous thromboembolism (2 points), Vascular disease, Age 65–74 years, Sex category (female)]; INR = International normalized ratio; LMWH = low molecular weight heparin; DOAC = direct oral anticoagulant; OAC = oral anticoagulation; PCI = percutaneous coronary intervention; TIA = transient ischemic attack; TTR = time in therapeutic range; UFH = unfractionated heparin; VKA = vitamin K antagonist oral anticoagulant.

**Extended Data Table 5:**
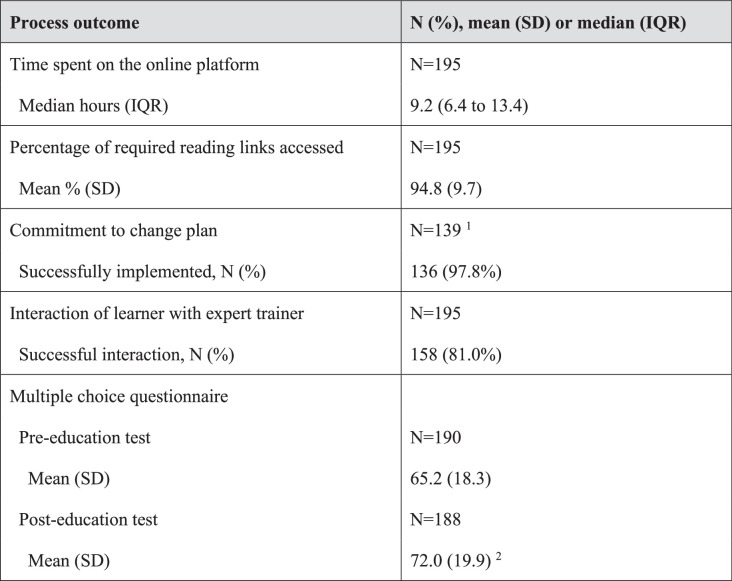
Process Outcomes in the Intervention Group

^1^56 learners were excluded from this analysis as they did not commit to at least two items, or there were missing data at baseline or follow-up. If all the excluded learners are assumed to have failed their commitment to change, then successful implementation is 69.7%.

^2^post-hoc comparison against pre-education test, p < 0.0001 (2-sided paired t-test not adjusted for multiple comparisons).

**Extended Data Table 6:**
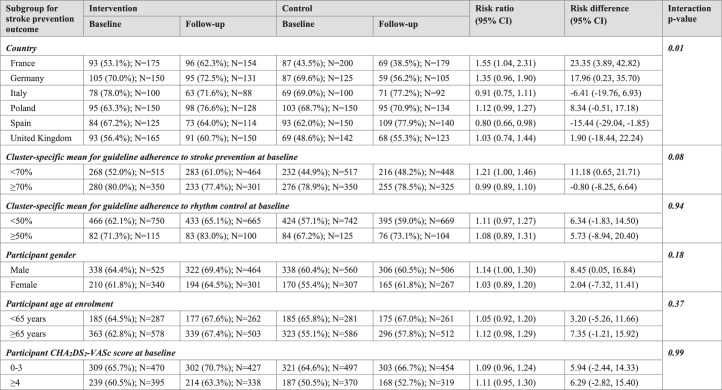
Subgroup Analyses on Stroke Prevention

CHA_2_DS_2_-VASc = stroke/thromboembolism risk score [Congestive heart failure, Hypertension, Age ≥75 years (2 points), Diabetes mellitus, Stroke or previous thromboembolism (2 points), Vascular disease, Age 65–74 years, Sex category (female)].

**Extended Data Table 7:**
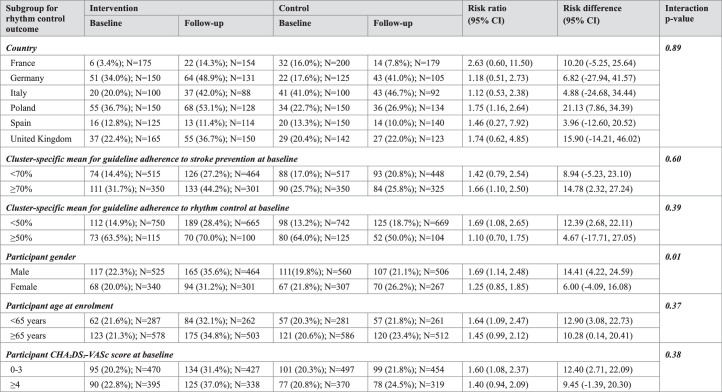
Subgroup Analyses on Rhythm Control

CHA_2_DS_2_-VASc = stroke/thromboembolism risk score [Congestive heart failure, Hypertension, Age ≥75 years (2 points), Diabetes mellitus, Stroke or previous thromboembolism (2 points), Vascular disease, Age 65–74 years, Sex category (female)].

**Extended Data Table 8:**
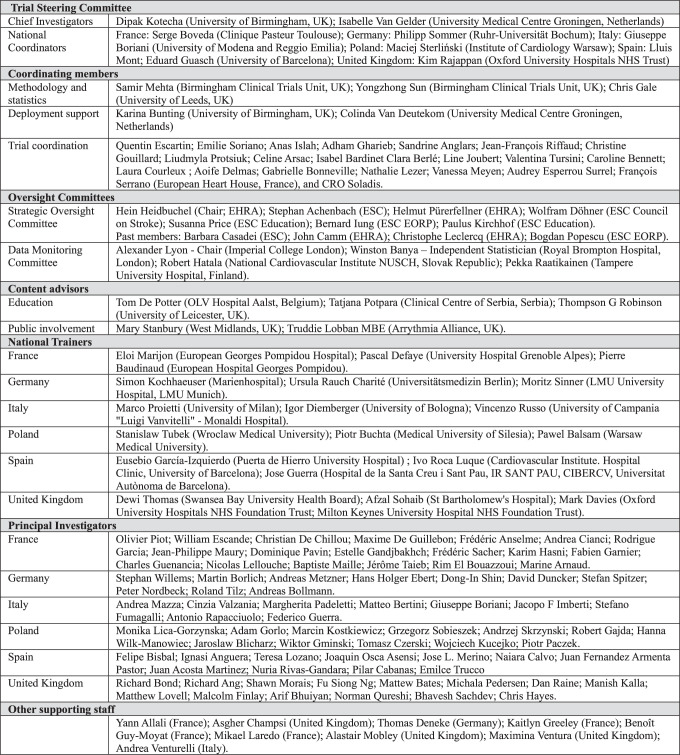
STEEER-AF Central Teams

EORP = EURObservational Research Programme; EHRA = European Heart Rhythm Association; ESC = European Society of Cardiology.

**Extended Data Table 9:**
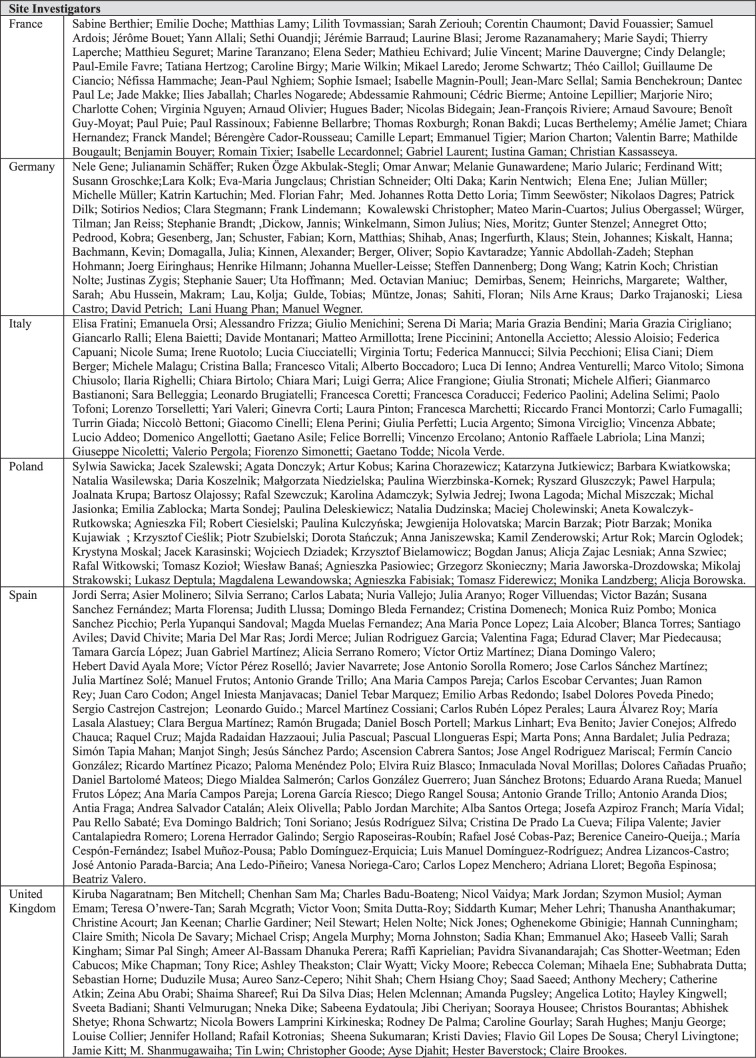
STEEER-AF Local Teams

## Data Availability

Anonymized summary data will be made available for noncommercial purposes on request to the corresponding author, after completion and publication of clinical follow-up and secondary paper (D.K.; d.kotecha@bham.ac.uk; 90-day response time for decisions following review by the STEEER-AF trial management group).
