# Supplementary material for: Education of healthcare professionals to improve guideline adherence in atrial fibrillation: the STEEER-AF cluster-randomized clinical trial
Source: Nat Med. 2025 Jun 13;31(8):2647–54. doi: 10.1038/s41591-025-03751-2 (PMC12353803; doi:10.1038/s41591-025-03751-2)
Supplement: Supplementary file 1 — CONSORT cluster checklist, trial protocol and statistical analysis plan. [file 41591_2025_3751_MOESM1_ESM.pdf]

nature medicine

Supplementary information https://doi.org/10.1038/s41591-025-03751-2

# Education of healthcare professionals to improve guideline adherence in atrial fibrillation: the STEEER-AF cluster-randomized clinical trial

In the format provided by the authors and unedited

# CONSORT cluster trial extension checklist: STEEER-AF trial

**CONSORT 2010 checklist of information to include when reporting a cluster randomised trial**

| Section/topic and item No | Standard checklist item | Extension for cluster designs | Page No* |
|---|---|---|---|
| **Title and abstract** | | | |
| 1a | Identification as a randomised trial in the title | Identification as a cluster randomised trial in the title | 1 |
| 1b | Structured summary of trial design, methods, results, and conclusions (for specific guidance see CONSORT for abstracts)[11 12] | See table 2 | 3 |
| **Introduction** | | | |
| Background and objectives: | | | |
| 2a | Scientific background and explanation of rationale | Rationale for using a cluster design | 4 |
| 2b | Specific objectives or hypotheses | Whether objectives pertain to the cluster level, the individual participant level, or both | 4 |
| **Methods** | | | |
| Trial design: | | | |
| 3a | Description of trial design (such as parallel, factorial) including allocation ratio | Definition of cluster and description of how the design features apply to the clusters | 22,23 |
| 3b | Important changes to methods after trial commencement (such as eligibility criteria), with reasons | | no changes 22 |
| Participants: | | | |
| 4a | Eligibility criteria for participants | Eligibility criteria for clusters | 22,23 |
| 4b | Settings and locations where the data were collected | | 22,23 |
| Interventions: | | | |
| 5 | The interventions for each group with sufficient details to allow replication, including how and when they were actually administered | Whether interventions pertain to the cluster level, the individual participant level, or both | 24 & design paper |
| Outcomes: | | | |
| 6a | Completely defined prespecified primary and secondary outcome measures, including how and when they were assessed | Whether outcome measures pertain to the cluster level, the individual participant level, or both | 24,25 |
| 6b | Any changes to trial outcomes after the trial commenced, with reasons | | no changes |
| Sample size: | | | |
| 7a | How sample size was determined | Method of calculation, number of clusters(s) (and whether equal or unequal cluster sizes are assumed), cluster size, a coefficient of intracluster correlation (ICC or *k*), and an indication of its uncertainty | 25 |
| 7b | When applicable, explanation of any interim analyses and stopping guidelines | | protocol plus design paper |
| **Randomisation** | | | |
| Sequence generation: | | | |
| 8a | Method used to generate the random allocation sequence | | 23 |
| 8b | Type of randomisation; details of any restriction (such as blocking and block size) | Details of stratification or matching if used | 23 |
| Allocation concealment mechanism: | | | |
| 9 | Mechanism used to implement the random allocation sequence (such as sequentially numbered containers), describing any steps taken to conceal the sequence until interventions were assigned | Specification that allocation was based on clusters rather than individuals and whether allocation concealment (if any) was at the cluster level, the individual participant level, or both | 23 |
| Implementation: | | | |
| 10 | Who generated the random allocation sequence, who enrolled participants, and who assigned participants to interventions | Replaced by 10a, 10b, and 10c | |
| 10a | | Who generated the random allocation sequence, who enrolled clusters, and who assigned clusters to interventions | 23 |

| Section/topic and item No | Standard checklist item | Extension for cluster designs | Page No* |
|---|---|---|---|
| 10b | | Mechanism by which individual participants were included in clusters for the purposes of the trial (such as complete enumeration, random sampling) | 22,23 |
| 10c | | From whom consent was sought (representatives of the cluster, or individual cluster members, or both) and whether consent was sought before or after randomisation | 23 |
| Blinding: | | | |
| 11a | If done, who was blinded after assignment to interventions (for example, participants, care providers, those assessing outcomes) and how | | 23 |
| 11b | If relevant, description of the similarity of interventions | | n/a |
| Statistical methods: | | | |
| 12a | Statistical methods used to compare groups for primary and secondary outcomes | How clustering was taken into account | 25,26 |
| 12b | Methods for additional analyses, such as subgroup analyses and adjusted analyses | | 25,26 |
| **Results** | | | |
| Participant flow (a diagram is strongly recommended): | | | |
| 13a | For each group, the numbers of participants who were randomly assigned, received intended treatment, and were analysed for the primary outcome | For each group, the numbers of clusters that were randomly assigned, received intended treatment, and were analysed for the primary outcome | 5, Fig1 |
| 13b | For each group, losses and exclusions after randomisation, together with reasons | For each group, losses and exclusions for both clusters and individual cluster members | Fig1 |
| Recruitment: | | | |
| 14a | Dates defining the periods of recruitment and follow-up | | 5 |
| 14b | Why the trial ended or was stopped | | n/a |
| Baseline data: | | | |
| 15 | A table showing baseline demographic and clinical characteristics for each group | Baseline characteristics for the individual and cluster levels as applicable for each group | 12 |
| Numbers analysed: | | | |
| 16 | For each group, number of participants (denominator) included in each analysis and whether the analysis was by original assigned groups | For each group, number of clusters included in each analysis | Fig 1<br>Ext table 1-3 |
| Outcomes and estimation: | | | |
| 17a | For each primary and secondary outcome, results for each group, and the estimated effect size and its precision (such as 95% confidence interval) | Results at the individual or cluster level as applicable and a coefficient of intracluster correlation (ICC or *k*) for each primary outcome | 5,6,<br>Table 2 |
| 17b | For binary outcomes, presentation of both absolute and relative effect sizes is recommended | | 5,6,<br>Table 2 |
| Ancillary analyses: | | | |
| 18 | Results of any other analyses performed, including subgroup analyses and adjusted analyses, distinguishing prespecified from exploratory | | 6,7<br>Ext data tables |
| Harms: | | | |
| 19 | All important harms or unintended effects in each group (for specific guidance see CONSORT for harms106) | | n/a |
| **Discussion** | | | |
| Limitations: | | | |
| 20 | Trial limitations, addressing sources of potential bias, imprecision, and, if relevant, multiplicity of analyses | | 9, 10 |
| Generalisability: | | | |
| 21 | Generalisability (external validity, applicability) of the trial findings | Generalisability to clusters and/or individual participants (as relevant) | 7,8 |

| Section/topic and item No | Standard checklist item | Extension for cluster designs | Page No* |
|---|---|---|---|
| Interpretation: | | | |
| 22 | Interpretation consistent with results, balancing benefits and harms, and considering other relevant evidence | | 7,8,9 |
| **Other information** | | | |
| Registration: | | | |
| 23 | Registration number and name of trial registry | | 22 |
| Protocol: | | | |
| 24 | Where the full trial protocol can be accessed, if available | | supplied |
| Funding: | | | |
| 25 | Sources of funding and other support (such as supply of drugs), role of funders | | 11 |

*Page numbers optional depending on journal requirements.

## STEEER-AF concept paper:

KV Bunting, IC Van Gelder, D Kotecha. STEEER-AF: a cluster-randomized education trial from the ESC. Eur Heart J. 2020;41:1952-1954. https://doi.org/10.1093/eurheartj/ehaa421.

## STEEER-AF design paper (including algorithms):

M Sterlinski, KV Bunting, G Boriani, S Boveda, E Guasch, L Mont, K Rajappan, P Sommer, S Mehta, Y Sun, CP Gale, C van Deutekom, IC Van Gelder, D Kotecha. Design and deployment of the STEEER-AF trial to evaluate and improve guideline adherence: a cluster-randomized trial by the European Society of Cardiology and European Heart Rhythm Association. Europace. 2024;26: https://doi.org/10.1093/europace/euae178.

DocuSign Envelope ID: 1B8B2092-5DB2-4866-899B-414B5B6B56FC

# STEEER-AF Cluster Randomized Trial Protocol

**Stroke prevention and rhythm control Therapy: Evaluation of an Educational Programme of the European society of cardiology in a cluster-Randomised trial in patients with Atrial Fibrillation**

EudraCT number: 2020-000792-20

ClinicalTrials.org: NCT04396418

**Protocol version:**

**30 June 2020 (version 1.0)**

# Protocol History

| Protocol Amendments | | | | |
|---|---|---|---|---|
| The following amendments and/or administrative changes have been made to this protocol since the implementation of the first approved version: | | | | |
| Amendment number | Date of amendment | Protocol version number | Type of amendment | Summary of amendment(s) |
| | | | | |

DocuSign Envelope ID: 1B8B2092-5DB2-4866-899B-414B5B6B56FC

# Trial Synopsis

| | | |
|---|---|---|
| Title | **S**troke prevention and rhythm control **T**herapy: **E**valuation of an **E**ducational programme of the **E**uropean society of cardiology in a cluster-**R**andomised trial in patients with **A**trial **F**ibrillation (STEEER-AF) | |
| Primary objectives | To determine whether a comprehensive educational programme for healthcare professionals will increase the rate of appropriate stroke prevention and rhythm control therapy in patients with atrial fibrillation (AF) | |
| Study design | Prospective, parallel group, two-arm, unblinded, international, cluster-randomized controlled trial | |
| Randomised intervention | Intervention arm: A 16-week blended learning programme targeting stroke prevention and rhythm control therapy at the healthcare professional level, with controlled assessments, a commitment to change plan, and reinforcement actions<br>Control arm: No added education of healthcare professionals | |
| Population | Patients diagnosed with AF | |
| Cluster definition | A hospital or health centre | |
| Number of randomised clusters and patients | 70 centres with a cluster size of 25 patients; total estimated number of patients 1750 | |
| Number of countries | 6: France, Germany, Italy, Poland, Spain, United Kingdom | |
| Duration of study | 8-12 weeks patient recruitment period at each centre; 16 weeks educational intervention period; primary & secondary outcomes at 6-9 months post-randomisation; remote follow-up for clinical events (no new patient contact) at 18 months from randomisation | |
| Inclusion criteria | Cluster level: | Site agrees to participation, enrolment and follow-up of 25 patients, and randomisation of the site |
| | Patient level: | Diagnosed with AF and consents to data collection at baseline and follow-up |
| Exclusion criteria | Cluster level: | None |
| | Patient level: | Patients aged under 18 years of age, pregnant, planning pregnancy or breastfeeding, participating in another clinical trial of an investigational medicinal product or device, or with a life expectancy of less than 2 years |
| Patient assessment | Baseline (time of recruitment) and at follow-up routine appointment (6-9 months) | |
| Co-primary outcomes | Full adherence to class I and III guidelines for stroke prevention<br>Full guideline-adherence to class I and III for rhythm control therapy<br>(based on the European Society of Cardiology AF Guidelines) | |
| Secondary outcomes | Proportion of relevant guidelines adhered to for stroke prevention<br>Proportion of relevant guidelines adhered to for rhythm control | |

DocuSign Envelope ID: 1B8B2092-5DB2-4866-899B-414B5B6B56FC

| | |
|---|---|
| | Proportion of patients treated with oral anticoagulants including both class I and II guideline indications<br>Proportion of patients treated with oral anticoagulants according to class I guideline indications<br>Integrated AF management approach (education, lifestyle support, self-management, shared decision making, support tools, adherence and multidisciplinary management)<br>Patient-reported quality of life using the EQ-5D-5L questionnaire |
| Process outcomes (intervention arm only) | Improvement in knowledge and guideline-adherent practice by healthcare professionals using the educational intervention |
| Remote follow-up | Major clinical events during the 18-month period after randomisation (composite of all-cause mortality, non-fatal stroke, transient ischaemic attack, pulmonary embolus, systemic embolic event, acute coronary syndrome, myocardial infarction, heart failure, and major/clinically-relevant bleeding; and hospital admission) |
| Sample size and Power | Based on published surveys, 80% of control patients are expected to be guideline-adherent for the stroke prevention outcome. We aim to detect a clinically important relative difference of 10% (88% outcome in the intervention arm), and assume a conservative intracluster correlation coefficient of 0.04. Accounting for 10% loss to follow-up, a sample size of 1750 patients across 70 centres provides 85% power (2-sided alpha 0.05). For the rhythm control outcome, this sample size provides 85% power to detect an increase in guideline-adherence from 50% to 61% |
| Trial Management | A Strategic Oversight Committee and Trial Steering Committee will provide overall oversight, supported by an independent Data Monitoring Committee. The ESC coordinator and National Coordinators will coordinate sites within their country and represent clusters to the Steering Committee |
| Sponsor | The European Society of Cardiology (ESC) |
| Funding | STEEER-AF is funded by the ESC through unrestricted educational grants from industry, and is a joint effort of the European Heart Rhythm Association, the ESC Education Committee and the ESC Council on Stroke. |

# Trial Team

| Trial Steering Committee | |
|---|---|
| Chief Investigator | Dipak Kotecha (University of Birmingham, UK) |
| Co-Chief Investigator | Isabelle van Gelder (University Medical Centre Groningen, Netherlands) |
| Stroke and Rhythm Leads | Tom De Potter (OLV Hospital Aalst, Belgium)<br>Tatjana Potpara (Clinical Centre of Serbia, Serbia)<br>Thompson G Robinson (University of Leicester, UK) |
| National Coordinators | France: Serge Boveda (Clinique Pasteur)<br>Germany: Philipp Sommer (Ruhr-Universität Bochum)<br>Italy: Giuseppe Boriani (University of Modena and Reggio Emilia)<br>Poland: Maciej Sterliński (Institute of Cardiology Warsaw)<br>Spain: Luis Mont (University of Barcelona)<br>United Kingdom: Kim Rajappan (University of Oxford) |
| Randomisation and Statistics | Pollyanna Hardy (Birmingham Clinical Trials Unit, UK)<br>Samir Mehta (Birmingham Clinical Trials Unit, UK) |
| Trial coordination ESC | Caroline Bennett (European Heart House, France)<br>Patti-Ann McNeill (European Heart House, France) |
| Oversight Committees | |
| Strategic Oversight Chair | Hein Heidbuchel (EHRA President) |
| Strategic Oversight Committee | Barbara Casadei (ESC President)<br>John Camm (EHRA Past President)<br>Wolfram Döhner (ESC Council on Stroke Chair)<br>Paulus Kirchhof (ESC Education Committee Chair)<br>Chris Gale (EURObservational Research Programme Chair; non-voting)<br>Isabel Bardinet (ESC Chief Executive Officer; non-voting)<br>Dipak Kotecha (Chief Investigator; non-voting)<br>Isabelle van Gelder (Co-Chief Investigator; non-voting) |
| Data Monitoring Committee | Alex Lyon - Chair (Imperial College London)<br>Winston Banya – Independent Statistician (Royal Brompton Hospital, London)<br>Robert Hatala (National Cardiovascular Institute NUSCH, Slovak Republic)<br>Pekka Raatikainen (Tampere University Hospital, Finland) |

DocuSign Envelope ID: 1B8B2092-5DB2-4866-899B-414B5B6B56FC

# Signature Page

*This protocol has been approved by: Chief Investigator*

Name: Dipak Kotecha

Signature: DocuSigned by: Dipak Kotecha 16CA812605D147D...

Date: 7/20/2020

*Sponsor (ESC)*

Name: Chris P Gale

Chairman of EURObservational Research Programme (EORP)

Signature: DocuSigned by: Chris P Gale 1473255866EF480...

Date: 7/9/2020

*Sponsor (ESC)*

Name: Céline Arsac

EORP Team Manager

Signature: DocuSigned by: Céline Arsac 16E53C233112439...

Date: 7/15/2020

*Statistician*

Name: Samir Mehta

Signature: DocuSigned by: Samir Mehta D85AAD712ABC4F6...

Date: 7/9/2020

DocuSign Envelope ID: 1B8B2092-5DB2-4866-899B-414B5B6B56FC

# Investigator Signature Page

I have read and agree to the protocol, as detailed in this document. I agree to adhere to the protocol as outlined and in accordance with approval from the Research Ethics Committee and any relevant regulatory authorities.

I am aware of my responsibilities as an Investigator under the guidelines of Good Clinical Practice (GCP), the Declaration of Helsinki, local regulations (as applicable) and the trial protocol, and I agree to conduct the trial according to these guidelines and to appropriately direct and assist the staff under my control, who will be involved in the trial.

Trial Name: STEEER-AF

Protocol Version Number: Version: __.__

Protocol Version Date: __ __ / __ __ __ / __ __ __ __

Site Name: ___________________________________

Investigator Name: ___________________________________

Signature: ___________________________________

Date: __ __ / __ __ __ / __ __ __ __

DocuSign Envelope ID: 1B8B2092-5DB2-4866-899B-414B5B6B56FC

# Abbreviations

| | |
|---|---|
| AF | Atrial fibrillation |
| CRM | Cluster Representation Mechanism |
| DMC | Data Monitoring Committee |
| EHRA | European Heart Rhythm Association |
| ESC | European Society of Cardiology |
| NOAC | Non-Vitamin K antagonist oral anticoagulant |
| NC | National Coordinator |
| PI | Principal Investigator (PI) |
| STEEER-AF | Stroke prevention and rhythm control Treatment: Evaluation of an Educational programme of the European society of cardiology in a cluster-Randomised trial in patients with Atrial Fibrillation |
| SOC | Strategic Oversight Committee |
| TSC | Trial Steering Committee |

# Acknowledgments

We wish to thank the Patient Involvement Team for their assistance in designing this trial to meet the needs of patients with atrial fibrillation and writing the patient information leaflet: Mary Stanbury (patient representative), Jaqueline Jones (public representative) and Trudie Lobban (Heart Rhythm Alliance/AF patient association).

STEEER-AF was designed with the assistance of the ESC, EHRA and Stroke Council boards, the ESC and EHRA Scientific Review Committees, the ESC Education Committee and Liberum Independent Medical Education (London, UK). We also wish to express thanks to the ESC staff (European Heart House, Sophia Antipolis, France) and Colin Baigent (external methodology reviewer, Oxford, UK).

We are grateful to Boehringer Ingelheim, BMS/Pfizer Alliance, Bayer, Daiichi Sankyo and Boston Scientific for providing unrestricted educational grants to the ESC to help fund this trial. We also acknowledge support from the Oxford Biomedical Research Centre, Oxford, UK, funded by the National Institute for Health Research (NIHR). The views expressed in this protocol are those of the authors and not necessarily those of the NIHR, UK Department of Health, or other funders listed.

DocuSign Envelope ID: 1B8B2092-5DB2-4866-899B-414B5B6B56FC

# Table of Contents


**Protocol History** 2
**Trial Synopsis** 3
**Trial Team** 5
**Signature Page** 6
**Investigator Signature Page** 7
**Abbreviations** 8
**Acknowledgments** 8
**Table of Contents** 9
**1. Introduction and Rationale** 11
1.1 Burden of atrial fibrillation 11
1.2 Guideline-adherent care 11
1.3 Stroke prevention in atrial fibrillation 11
1.4 Rhythm control and integrated management 12
1.5 Educational approaches to improve outcomes 12
**2. Aims and Objectives** 13
2.1 Primary objective 13
2.2 Primary hypothesis 13
2.3 Secondary objectives 14
2.4 Secondary hypothesis 14
**3. Study Design** 14
3.1 Trial design and Study setting 14
3.2 Cluster definition and Study sites 14
3.3 Process of site and learner selection 14
3.4 Patient visits 15
3.5 Remote follow-up 15
**4. Study Flowcharts** 15
Figure 1: STEEER-AF cluster-level overview 15
Figure 2: STEEER-AF patient-level overview 16
**5. Eligibility Criteria** 17
5.1 Inclusion criteria 17
5.2 Exclusion criteria 17
**6. Intervention** 17
6.1 Educational intervention 17
Table 1: Educational intervention plan 18
6.2 Control group 18
6.3 Criteria for discontinuing or modifying allocated interventions 18
6.4 Strategies to improve adherence 19
6.5 Concomitant care/interventions permitted or prohibited 19
6.6 Benefit-Risk Evaluation 19
**7. Outcomes** 20
7.1 Co-primary outcomes 20

7.2 Secondary outcomes ....... 20
7.3 Process outcomes ....... 21
7.4 Remote follow-up ....... 21
**8. Randomisation and Blinding ....... 22**
**9. Data Collection ....... 23**
9.1 Data capture at centre level ....... 23
9.2 Data capture at patient level ....... 23
9.3 Healthcare professional improvement in knowledge ....... 23
9.4 Data management ....... 24
9.5 Ascertainment of outcomes ....... 24
9.6 Serious Adverse Events ....... 25
**10. Statistical methods ....... 25**
10.1 Sample size ....... 25
10.2 Analysis ....... 25
10.3 Primary outcomes ....... 26
10.4 Other outcomes ....... 26
**11. Trial Oversight ....... 27**
11.1 Trial Steering Committee ....... 27
11.2 Strategic Oversight Committee ....... 27
11.3 Data Monitoring Committee ....... 27
**12. Consent ....... 28**
12.1 Cluster level ....... 28
12.2 Patient level ....... 28
**13. Ethics and Regulatory Requirements ....... 28**
13.1 Ethical principles ....... 28
13.2 Ethical Approval ....... 29
13.3 Confidentiality and Data Protection ....... 29
13.4 Insurance and Indemnity ....... 29
13.5 Statement of Compliance ....... 30
**14. Investigators, Disclosures and Dissemination ....... 30**
14.1 Investigators and financial disclosures ....... 30
14.2 Dissemination and publication plan ....... 30
14.3 Protocol and publication adherence ....... 30
**15. References ....... 31**

**Appendix 1 ....... 33**
**Appendix 2 ....... 34**

DocuSign Envelope ID: 1B8B2092-5DB2-4866-899B-414B5B6B56FC

# 1. Introduction and Rationale

## 1.1 Burden of atrial fibrillation

Atrial fibrillation (AF) is common, will continue to become more prevalent in the years to come, and is associated with considerable burden to both patients and healthcare services.[1, 2] Healthcare professionals that manage patients with AF are faced with numerous challenges to provide care that improves the outcomes of patients with AF, including therapies for stroke prevention and rhythm control.[3] These two facets of AF management are complex, relying on integration of patient-level factors, locally-available treatment strategies and appropriate decision-making by physicians. Hence, the burden of AF extends to the education and knowledge-base of those treating patients with AF.

## 1.2 Guideline-adherent care

The European Society of Cardiology (ESC) provides practice guidelines for a number of cardiovascular conditions in association with the European Heart Rhythm Association (EHRA).[1] Guideline-adherent care is defined as the implementation of guideline recommendations to individual patients. Guideline-adherent treatment has been shown to improve the outcomes of patients with AF, including lower rates of mortality, incident stroke and major bleeding.[4-6] The challenge for healthcare services is that guidelines are often not applied, applied partially, or even incorrectly, with the education and training of healthcare workers identified as major barriers for guideline implementation.[7-9]

## 1.3 Stroke prevention in atrial fibrillation

The incidence of stroke varies between 100 and 400 per 100,000 patients per year, and at least a third of those with ischaemic stroke have either previously known or newly-detected AF at the time of diagnosis.[10] Stroke risk-stratification schemes for patients with AF are in common use to help clinicians choose which patients require oral anticoagulants to prevent stroke and thromboembolism.[1] In general, AF patients without other stroke risk factors do not need anticoagulants (and neither should they be prescribed aspirin). In contrast, patients with stroke risk factors have a net benefit from anticoagulants, beyond the main risk of these drugs such as intracerebral haemorrhage and other major bleeding. Thus, a decision on whether or not to start anticoagulants is made considering the balance of expected stroke risk reduction and bleeding risk, in the context of informed patient preferences and values. Stroke and bleeding risk factors overlap, but rather than withholding anticoagulants, a high bleeding risk should prompt the correction of modifiable factors to increase the safety of anticoagulants for stroke prevention.[1]

Oral anticoagulation is often inadequately managed due to deficient adherence to AF guidelines[11], with varying rates of guideline adherence across and within European countries.[12] Multidisciplinary approaches to guideline-

DocuSign Envelope ID: 1B8B2092-5DB2-4866-899B-414B5B6B56FC

based care have been piloted and have shown success[13], but integrated AF clinics have yet to be widely implemented, in part because they require dedicated nurses and cardiologists with considerable logistical infrastructure. Although there has been a rapid uptake in the use of non-Vitamin K oral anticoagulants (NOACs) in place of warfarin, a large proportion of patients remain undertreated[14], or are mistakenly given antiplatelet drugs[15], which increase the risk of bleeding but do not adequately prevent stroke. There are also issues with patients who may or may not have an indication for concomitant antiplatelet therapy, and those with a contraindication to oral anticoagulation who should be considered for alternative methods of stroke prevention, such as left atrial appendage exclusion. Many of these decisions are complex and require a well-educated workforce to achieve the best patient outcomes.[16]

## 1.4 Rhythm control and integrated management

Rhythm control in patients with AF aims to restore and maintain sinus rhythm. Although many clinicians believe that maintaining sinus rhythm improves outcomes in AF patients, trials have generally showed no reduction in adverse clinical outcomes with rhythm control compared to heart rate control, or with interventional compared to medical therapy alone.[17] Therefore, the current rationale for rhythm control therapy is to improve symptoms in AF patients who remain symptomatic on adequate rate control therapy.[1]

A number of different options are available for rhythm control, including electrical cardioversion, antiarrhythmic drugs, endocardial catheter ablation of pulmonary veins, epicardial thoracoscopic ablation, and open-heart maze surgery, as well as hybrid approaches with a mix of these strategies. There remain major challenges for clinicians to choose whether or not to institute rhythm control in individual patients, and also what technique to use. To adequately implement guidelines on rhythm control based on the current evidence-base, a structured and multidisciplinary team-based approach is required. This should include education and cooperation of general practitioners, cardiologists, cardiac surgeons, stroke neurologists and allied AF health practitioners.[3] In addition, it is vital to consider patient support and education.[18]

## 1.5 Educational approaches to improve outcomes

Recently, EHRA and the ESC performed a multinational educational needs assessment study of cardiologists, general practitioners and neurologists, in order to evaluate the knowledge and skills of physicians caring for AF patients.[19] Many physicians reported insufficient skills to use stroke risk assessment for management decisions, and only around 60% systematically calculated risk scores for their patients. There was considerable uncertainty on how to deal with anticoagulant therapy in complex patients, as well as the impact of comorbidities such as renal dysfunction or gastric pathology. There was also a high disparity in the use of rate and rhythm control strategies, with 33% of cardiologists admitting insufficient knowledge to assess the indication for rhythm control, and 61% reporting inadequate skills to appropriately select patients for AF ablation.[19] This study identified that gaps in knowledge and skills of healthcare professionals is a major factor in poor adherence to

guidelines. Targeted educational programmes are therefore an attractive approach to close knowledge gaps, facilitate the correct implementation of trial-based treatments, and could contribute to improved outcomes in AF patients. The IMPACT-AF trial demonstrated that physician education can improve treatment prescription at a patient level, although this cluster randomised trial was limited to assessment of oral anticoagulation.[20]

EHRA/ESC accept that further improvement in patient outcomes for AF will require a broader approach to all aspects of AF management, with a concerted effort to educate a variety of healthcare professionals and provide specific guideline-related training and support.[21] Contemporary educational programmes require a multi-faceted and multi-modality approach to make best use of digital learning opportunities[18], and EHRA/ESC also provide a wider context for education including both traditional and online learning methods.[16] However, the true value of these educational approaches is unknown. Considering the time and expense required to train a workforce and keep this body of clinical staff updated, a formal evaluation of the effectiveness of AF education is warranted. Randomised controlled trials provide the highest level of evidence[22], and a cluster-randomised approach is a suitable design for testing an educational programme where contamination of effect could occur across neighbouring individual patients.[23]

This protocol outlines the design of the STEEER-AF trial (**S**troke prevention and rhythm control **T**reatment: **E**valuation of an **E**ducational programme of the European society of cardiology in a cluster-**R**andomised trial in patients with **A**trial **F**ibrillation) – a joint effort by the ESC Education team, EHRA and the ESC Council on Stroke. STEEER-AF is a pragmatic, cluster-randomised evaluation of a blended learning intervention designed to address these unmet educational needs for healthcare professionals, with the aim of improving class I and class III guideline-adherent therapy for patients with AF.

# 2. Aims and Objectives

## 2.1 Primary objective

To determine whether a comprehensive and structured educational programme for healthcare professionals treating patients with AF, compared to no added education, will improve guideline-adherent provision of patient-level treatments relating to stroke prevention and rhythm control.

## 2.2 Primary hypothesis

| | |
|---|---|
| Null hypothesis: (primary outcomes) | No difference in rates of appropriate stroke prevention or rhythm control treatments at patient-level, comparing the structured educational programme to no added education of healthcare professionals. |

### 2.3 Secondary objectives

To determine whether a comprehensive and structured educational programme for healthcare professionals treating patients with AF, compared to no added education, will improve the proportion of guidelines implemented at patient-level and the provision of integrated care, lead to benefit in patient-reported quality of life and reduce major adverse clinical outcomes.

### 2.4 Secondary hypothesis

| | |
|---|---|
| Null hypothesis:<br>(secondary outcomes) | No difference in the proportion of relevant guideline recommendations attained for stroke prevention or rhythm control, rates of integrated AF management, patient quality of life and clinical outcomes during follow-up at patient-level, comparing the structured educational programme to no added education of healthcare professionals. |

## 3. Study Design

### 3.1 Trial design and Study setting

Design: Pragmatic, international, multi-centre, parallel group, two-arm, unblinded, cluster-randomized controlled trial.

Setting: Hospitals and health centres in six European countries (France, Germany, Italy, Poland, Spain and the United Kingdom).

### 3.2 Cluster definition and Study sites

Individual hospitals and health centres in these six countries will be the clusters (defined as a site recruiting patients). See **Figure 1** for the cluster-level overview.

### 3.3 Process of site and learner selection

Sites in each country will be asked to contribute by their respective National Coordinators (NCs). Sites will encompass a broad range of centres across each country that treat patients with AF. The NC will identify a suitable cardiology physician in each centre to volunteer to manage STEEER-AF locally (the PI). They will nominate around 15 potential healthcare professionals in their institution that could benefit from the educational training programme, encompassing a range of multidisciplinary staff (the Investigators).

### 3.4 Patient visits

STEEER-AF is a pragmatic clinical trial and patients will be recruited by opportunity within the recruitment period for each centre (target 25 patients). Consecutive patients meeting the inclusion criteria and consenting under the care of Investigators will form the cohort of patients recruited within each centre. Data will be collected during the clinical visit to form the baseline assessment. Patient recruitment must be completed before a site is eligible for randomisation to either the educational intervention for healthcare professional learners, or control. Follow-up data will be acquired during the routine clinical follow-up of the patient at approximately 6-9 months post-randomisation for primary and secondary outcomes (in most cases, at least 2 months after completion of the educational programme in intervention centres). See **Figure 2** for patient-level overview.

### 3.5 Remote follow-up

An additional remote acquisition of clinical events will take place at 18 months, but this will not require a patient visit. PIs will be asked to collate clinical events from routine healthcare data and input this pseudonymised data into a predefined electronic case report form.

## 4. Study Flowcharts

**Figure 1: STEEER-AF cluster-level overview**

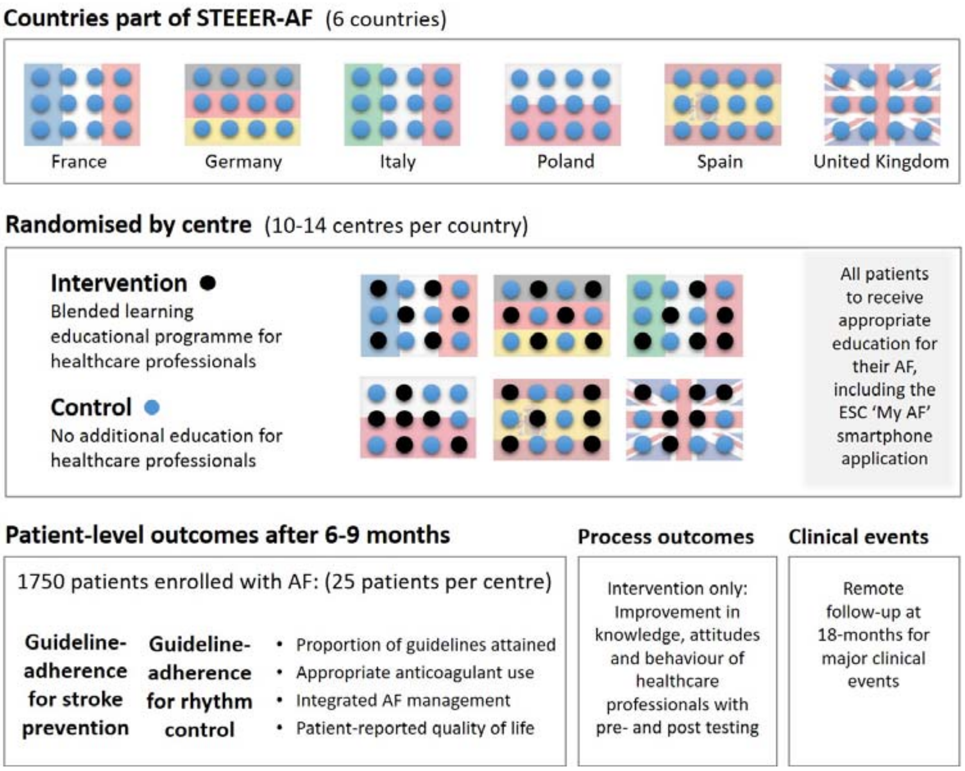

**Figure 2: STEEER-AF patient-level overview**

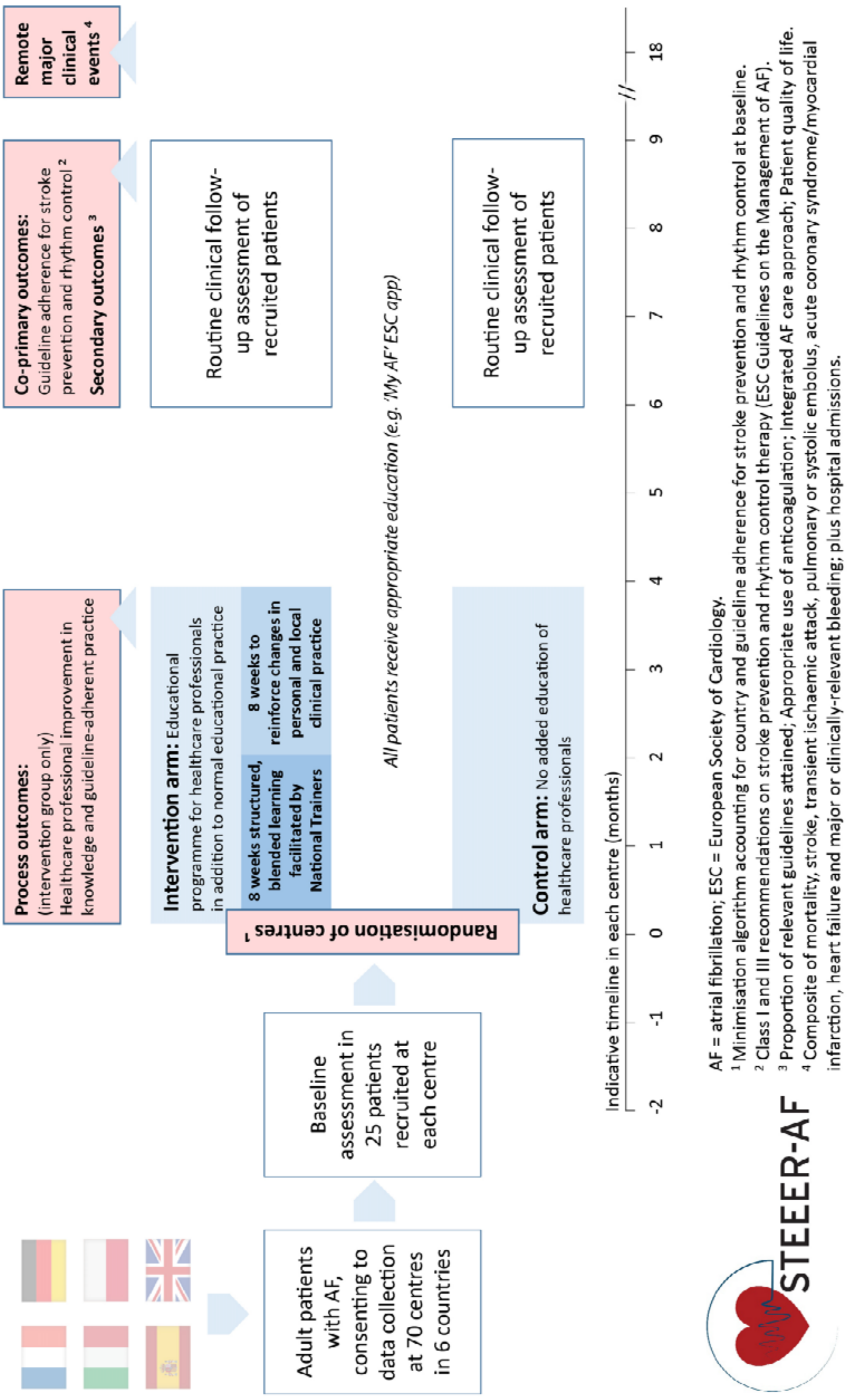

# 5. Eligibility Criteria

## 5.1 Inclusion criteria

Cluster level: Site agrees to participation, enrolment and follow-up of 25 patients, and randomisation of the site to intervention or control for healthcare practitioners at that centre.

Patient level: Diagnosed with AF based on ESC Guideline criteria (i.e. AF documented by ECG).

Informed, signed consent for data collection at baseline and follow-up.

## 5.2 Exclusion criteria

Cluster level: None.

Patient level: Patients aged under 18 years of age years.

Patients who are pregnant or planning to be pregnant, or who are breastfeeding at the time of consent.

Patients participating in another clinical trial of an investigational medicinal product or device.

Life expectancy of less than 2 years.

# 6. Intervention

## 6.1 Educational intervention

The intervention is a comprehensive and structured educational programme designed by stakeholders from the ESC Education Committee, EHRA and ESC Council on Stroke for healthcare professionals treating patients with AF. The programme is targeted towards stroke prevention and rhythm control, previously identified as key educational gaps.

The educational programme will operate using a “train-the-trainer” approach, whereby trained trainers will organise a country-specific training programme according to pre-defined learning outcomes in modules developed by the Trial Steering Committee. In brief**,** trainers in each country will be responsible for supervising and interacting online with Investigators in addition to face-to-face sessions (the Investigators are healthcare professionals who are involved in the care of AF patients at each centre). The educational programme will consist of 4 modules using a blended learning approach, each incorporating pre-reading, online resources, case-based peer interaction, in-person education, and evaluation of course materials and attained knowledge using pre and post-testing. Investigators at centres randomised to the intervention will be expected to devote 2-3 hours per

DocuSign Envelope ID: 1B8B2092-5DB2-4866-899B-414B5B6B56FC

week to the online components of the educational programme, with further clinically-relevant education according to the local needs of each learner. The programme will be submitted for accreditation, and participants will be able to claim a Continued Medical Education (CME) certificate after completion of the programme. A key part of the intervention process is generation of a locally-derived 'commitment to change' plan which will encompass elements that encourage and help a broad range of staff in each centre to manage patients with AF better (i.e. indirect benefits from the educational programme). These plans will include participation from the PI, and are expected to upskill staff at the centre and provide long-lasting improvements to patient care.

A plan of the intervention is presented in **Table 1**, broadly separated into knowledge and skills acquisition (active phase; first 2 months), followed by reinforcement activities and support for local effects of the intervention (next 2 months).

**Table 1: Educational intervention plan**

| Week | Educational intervention (total 16 week programme) |
|---|---|
| 1 | Consideration of local knowledge and needs, with quantitative pre-test for all learners |
| 2-6 | Blended learning experience, using a dedicated ESC platform for tailored learning, supported and moderated by the National Trainer. |
| 7 | Quantitative post-testing for all trainees. First draft of an individualised and local 'commitment to change' plan in discussion with the National Trainer and PI. |
| 8 | Face-to-face workshop with the National Trainer. Finalisation of 'commitment to change' plan. |
| 9-15 | Reinforcing activities and iterative feedback on actions to implement the 'commitment to change' plan. |
| 16 | Quantitative post-test for all trainees. |

## 6.2 Control group

The centres randomised to the control group will not receive the educational intervention, but will participate in data collection of patient-level data at baseline, follow-up at 6-9 months post-randomisation and follow-up at 18 months of remote clinical events.

## 6.3 Criteria for discontinuing or modifying allocated interventions

No pre-specified criteria for centre withdrawal are planned. If necessary, clusters may withdraw from the trial after discussion with the NC and Chief Investigator. Data collected up to that time-point may still be used in analysis.

DocuSign Envelope ID: 1B8B2092-5DB2-4866-899B-414B5B6B56FC

The structured educational intervention will be country-specific and adapted to local needs, including options for language selection.

## 6.4 Strategies to improve adherence

The Trial Steering Committee, through the ESC coordinator, will review average attendance at in-person educational events in the programme and NCs will encourage centres that demonstrate low attendance. Local PIs will be tasked with ensuring appropriate use of online materials, and progression in knowledge and skills (Table 1). Additional procedures for confirming local uptake of the educational programme include review of the implementation of the 'commitment to change' plan.

## 6.5 Concomitant care/interventions permitted or prohibited

The educational programme is targeted to healthcare professionals and does not contain material directed at patients. However all patients, regardless of which centre they receive treatment in, will be offered education about AF and their treatments at baseline. Patients will be informed about 'Afib Matters' (an EHRA-designed patient website) and 'My AF' (a smartphone and tablet application designed by the ESC for patients available free of charge).[18] Other tools are also available through patient support groups, ESC-related online content, and charities such as the British Heart Foundation, and all such educational material directed at patients are permitted in this trial. Similarly, there are no prohibitions on (additional) education of healthcare professionals in either intervention or control clusters.

Usual standard of care is expected for patients in both arms of the trial, according to local policies.

## 6.6 Benefit-Risk Evaluation

No risk is present for patients as the educational intervention is directed to the healthcare professional only; patients will receive standard of care according to their locality and country. The intervention is expected to provide a benefit to healthcare professionals looking after patients with AF (guideline-adherent optimal management) and also to other professionals at the site. Even in the case of a neutral effect of the intervention on guideline-adherent practice, no harm to patients will come from the additional education provided to healthcare professionals, as this information is based on class I and class III recommendations from the ESC practice guidelines on the management of AF, published in collaboration with the European Association for Cardio-Thoracic Surgery, EHRA and the European Stroke Organisation.[1]

DocuSign Envelope ID: 1B8B2092-5DB2-4866-899B-414B5B6B56FC

# 7. Outcomes

Co-primary and secondary outcomes are all measured at the 6-9 months post-randomisation follow-up visit.

## 7.1 Co-primary outcomes

1. **Guideline-adherence for stroke prevention therapy, based on class I and III recommendations of the ESC AF Guidelines.** This will be assessed as the proportion of patients whose therapy adheres fully with these recommendations. This includes the appropriate prescription of oral anticoagulation in patients with an elevated stroke risk score according to the $CHA_2DS_2$-VASc score, and appropriate dosing of anticoagulants by assessing time in therapeutic range for patients on vitamin K antagonists, or NOAC dose in relation to comorbidities. It will also consider inappropriate prescription of antiplatelet agents and/or oral anticoagulants in patients at low stroke risk according to the $CHA_2DS_2$-VASc score, that concomitant anticoagulant and antiplatelet use is limited to accepted indications and time periods, whether healthcare professionals stop anticoagulation inappropriately, and secondary stroke prevention strategies. See Appendix 1 for the full list of class I and III recommendations in the current ESC Guidelines.
2. **Guideline-adherence for rhythm control therapy, based on the class I and III recommendations of the ESC AF Guidelines.** This will be assessed as the proportion of patients whose therapy adheres fully with these recommendations. This includes the full range of clinical scenarios: symptom-directed approaches, acute cardioversion of AF and associated stroke prevention strategies, antiarrhythmic drug prescription corresponding to guideline recommendations in relation to associated heart disease, catheter ablation of AF, AF surgery, and rhythm control in specific populations with Wolff-Parkinson-White (WPW) syndrome and hypertrophic cardiomyopathy. See Appendix 2 for the full list of class I and III recommendations in the current ESC Guidelines.

## 7.2 Secondary outcomes

1. Proportion of guidelines adhered to for stroke prevention relevant to that patient, based on class I and III recommendations of the ESC AF Guidelines.
2. Proportion of guidelines adhered to for rhythm control relevant to that patient, based on class I and III recommendations of the ESC AF Guidelines.
3. Proportion of eligible patients treated with oral anticoagulants, including both class I and II guideline indications (women with $CHA_2DS_2$-VASc score 2 or above; men with $CHA_2DS_2$-VASc score 1 or above).
4. Proportion of patients treated with oral anticoagulants according to class I guideline indications (women with $CHA_2DS_2$-VASc score 3 or above; men with $CHA_2DS_2$-VASc score 2 or above).

5. Integrated AF management score, based on a score out of eight for each patient consisting of the following consensus-defined markers of good integrated care and a yes/no answer based on feedback of the clinical interaction:

   (1) Structured patient education provided on stroke prevention and oral anticoagulant therapy;

   (2) Advice and education on lifestyle and risk factors management given to patient; (3) Support provided to patients to make lifestyle changes (for example, referral for smoking cessation or weight loss); (4) Encouragement and empowerment of the patient for self-management; (5) Shared decision making approach used; (6) Use of technology tools for information on AF, OR use of a checklist for management and communication, OR use of clinical decision support tools; (7) Monitoring of adherence to therapy and effectiveness (for example, asking about adherence to anticoagulant therapy and INR testing);

   (8) Engagement of a multidisciplinary team in AF management (for example, including nurses, ancillary support staff, pharmacists, surgeons or other medical specialities).
6. Patient-reported quality of life using the EQ-5D-5L for a) overall mean index value and b) visual analogue scale.

## 7.3 Process outcomes

Improvement in knowledge and guideline-adherent practice for healthcare professionals will be determined in the intervention arm only:

1. Multiple choice questions following the learning objectives of each educational module included in the online intervention. All learning objectives have been written in accordance with Donald Moore's outcome levels and are therefore measurable to levels 3, 4 and 5. The multiple choice questions include classical best of five responses, or multiple true-false with either one or more correct responses. The assessment will occur at the start of each learner's educational programme (pre-test) and are duplicated at the end of the 16-week educational intervention (post-test).
2. Time spent on the online platform for each learner.
3. Percentage of required reading links clicked by each learner.
4. In-person interaction of the learner with the National Trainer.
5. Commitment to change plan to assess local change in clinical practice.

## 7.4 Remote follow-up

At 18 months from randomisation, PIs will be asked to collate information on major clinical events that have occurred for each patient during the time since their baseline visit. The primary remote outcome is the composite of all-cause mortality, non-fatal stroke, transient ischaemic attack, pulmonary embolus, systemic embolic event, acute coronary syndrome, myocardial infarction, hospitalisation for heart failure, and/or major and clinically-

DocuSign Envelope ID: 1B8B2092-5DB2-4866-899B-414B5B6B56FC

relevant non-major bleeding (defined as requiring a hospital admission). Secondary remote outcomes are the individual components of the primary list and hospital admissions (number and duration).

# 8. Randomisation and Blinding

Randomisation will be performed only after the centre has enrolled their allocation of patients and baseline data has been transferred to the Birmingham Clinical Trials Unit (BCTU). The NCs and Chief Investigators will make a joint decision on the allocation number for that centre based on recruitment at other centres in that country (overall target 25 patients per centre). Prior to randomisation, eligibility will be checked at both cluster and patient-level.

Centres will be randomised in a 1:1 ratio to either educational intervention or usual care. A minimisation algorithm will be used to ensure balance between the 2 groups over the following variables:

- Country
- Cluster-specific mean for class I and III guideline adherence to stroke prevention at baseline in enrolled participants (<70 and ≥70%)
- Cluster-specific mean for class I and III guideline adherence to rhythm control at baseline in enrolled participants (<50 and ≥50%).

The allocation will be generated by the statistician at BCTU, with blinding to the identity of the centres. The statistician will inform the trial coordinating centre at the ESC, who will then notify the PI and arrange for deployment of the educational programme to sites randomised to the intervention group. This local deployment will include generating personal login details for the nominated healthcare professional learners in that institution, and contact with the National Trainer responsible for that centre.

Due to the nature of the intervention, it is not possible to blind hospital or health centre staff to the randomised allocation. However, to minimise selection bias, centres will not be randomised and therefore not informed of their allocated intervention until they have recruited the required number of patients.

Further recruitment of patients into the study will not be undertaken once the centre has been made aware of their allocated group. As patients will be enrolled before the site is randomised, they will not be aware of the randomised allocation, although can be told at follow-up if they enquire.

# 9. Data Collection

## 9.1 Data capture at centre level

Hospital or health centre information will be captured with a single electronic case report completed by the PI prior to patient recruitment at baseline. This will include current facilities with regard to cardiology services, and centre size and type.

## 9.2 Data capture at patient level

**Clinical history and measurements:** Patients recruited into the study will have identical data points collected at baseline and 6-9-month follow-up by the PI (who is not a treating healthcare practitioner of recruited patients). These will be obtained using an electronic case report form (CRF) covering AF history, other medical history, clinical measurements and routine blood tests, assessment of bleeding risk, current therapy for stroke prevention and rate control, and previous or planned rhythm control medications and procedures. Relevant dosage of NOAC therapy will be collected to ensure appropriate use according to the ESC AF Guidelines.

**Ethnicity data**: The CRF will collect information about whether the patient is of African/African-American descent in order to correctly calculate the estimated glomerular filtration rate, as renal function is an important component of management decisions.

**Integrated AF management:** Score out of eight from the domains listed in section 8.2, including information obtained from the patient after the consultation using a paper CRF.

**Symptom class and quality of life:** Modified EHRA symptom class (1, 2a, 2b, 3, 4), with class 2b being a threshold for treatment decisions, derived by the treating healthcare practitioner; Patient-reported quality of life using the EQ-5D-5L questionnaire, including the visual analogue scale using a paper CRF.

**Major clinical events:** These will be collected in the CRF at 18 months after randomisation by the PI using electronic health records (local and national) and where required, communication with participants. Events to be captured include death, cause of death, stroke, transient ischaemic attack, pulmonary embolus, systemic embolic event, acute coronary syndrome, myocardial infarction, heart failure, major or clinically-relevant bleeding, and hospital admissions.

## 9.3 Healthcare professional improvement in knowledge

Testing of healthcare professionals enrolled in the educational intervention will occur before and after the 16-week programme using multiple choice questions, with responses collected through the online educational platform. The platform collects time spent on the online modules for each learner, in addition to whether required reading links are accessed. Data on the local commitment to change plan is captured through a survey at the end of the educational intervention to assess changes in clinical practice as a result of the intervention. If the

DocuSign Envelope ID: 1B8B2092-5DB2-4866-899B-414B5B6B56FC

participants have not successfully completed their commitments, they will be asked to reflect on their approaches and any barriers for future deployment using a mixed methods qualitative interview process in selected participants.

## 9.4 Data management

Full details on Data entry, Quality control, Audits, Data monitoring, and Security and storage will be available in a Data Management Plan, which will be validated by the ESC, the Steering Committee and the Contract Research Organisation (CRO).

Data pertaining to primary, secondary and remote outcomes will be managed by the CRO, data relating to the educational intervention will be managed by the ESC, and randomisation and statistical analysis will be managed by the University of Birmingham.

## 9.5 Ascertainment of outcomes

An algorithm will be created to match the case report form items for each patient at baseline and follow-up with the relevant class I and class III guideline recommendations. The logic algorithm will first assess the relevance of that guideline to the particular patient, and then identify if the conditions are met for adherence or non-adherence. For example, in the class III guideline "NOACs are not recommended in patients with mechanical heart valves or moderate-to-severe mitral stenosis", the algorithm would include only those patients with either a mechanical heart valve or moderate-to-severe mitral stenosis, and then determine if there was inappropriate use of NOACs based on the case report form responses. A similar automated process will occur for all class I and class III guideline recommendations to ensure that guidelines are applied to the correct patients.

For the co-primary outcomes, the end-point of interest is a binary variable indicating whether the patient's management is adherent to <u>all</u> class I and class III guidelines for stroke prevention and rhythm control respectively. For the secondary outcomes, this will be recorded as the proportion of class I and class III guidelines with adherence for stroke prevention and rhythm control relevant to that patient.

With regard to oral anticoagulation, "effective" anticoagulation also requires appropriate dosage.[1] For vitamin K antagonists, this will be assessed through time in therapeutic range (INR 2.0 to 3.0 in patients without valve replacement). For NOACs, this will be assessed using the guideline-stipulated doses and dose-reduction strategies based on the relevant randomised controlled trials: Apixaban 5mg twice daily with dose reduction to 2.5mg twice daily if two out of three indications: weight <60kg, age >80 years, serum creatinine >133mmol/L (or estimated creatinine clearance <30mL/min); Dabigatran 150 or 110mg twice daily with no pre-specified dose-reduction criteria; Edoxaban 60mg once daily with dose reduction to 30mg once daily if: weight <60kg, estimated creatinine clearance <50mL/min, or concomitant therapy with potent P-glycoprotein inhibitors; Rivaroxaban 20mg once daily with dose reduction to 15mg once daily if creatinine clearance <50mL/min.

DocuSign Envelope ID: 1B8B2092-5DB2-4866-899B-414B5B6B56FC

### 9.6 Serious Adverse Events

STEEER-AF is directed at healthcare professionals, with cluster randomisation of additional education to these practitioners based on published best-practice guidelines. As no intervention is directed at patient-level, serious adverse event (SAE) reporting will not take place (from the patient perspective, STEEER-AF is an observational cohort design).

However, the trial will still have oversight by a Data Monitoring Committee to ensure the safety of all patients and healthcare professionals taking part in the study (see Section 11.3).

# 10. Statistical methods

## 10.1 Sample size

Based on published surveys, 80% of control patients are expected to receive guideline-adherent care for stroke prevention.[15, 24] A relative increase of 10% (i.e. absolute increase of 8% from 80% to 88%) is considered by the trial team as a clinically-relevant improvement in guideline adherence for stroke prevention therapy. A global educational trial for stroke prevention used an intracluster correlation coefficient (ICC) of 0.02.[20] As a conservative measure, our sample size calculations are based on a higher ICC of 0.04.[25] With a 5% two-sided alpha, cluster size of 25 patients, 70 clusters in total, 1750 patients and this ICC of 0.04, STEEER-AF will have a power of 85% for this outcome. This includes 10% loss to patient follow-up and a small amount of variation in cluster size (or loss of clusters), with an assumed coefficient of variation of cluster size of 0.20.

For the rhythm control co-primary outcome, estimates of the control group rate are 50%.[15, 26] With the same assumptions as above, the sample size would provide 85% power to detect an absolute increase in guideline-adherence for rhythm control from 50% to 61%.

## 10.2 Analysis

A separate Statistical Analysis Plan will be produced and will provide a comprehensive description of the planned statistical analyses. A brief outline of these analyses is given below. The primary comparison groups will be composed of patients treated in centres receiving the intervention, versus patients in centres receiving no intervention. In the first instance, all analyses will be based on the intention to treat principle, i.e. all clusters and participants will be analysed in the intervention group to which they were randomised, irrespective of protocol deviations.

## 10.3 Primary outcomes

For the co-primary outcomes, risk ratios and associated 95% confidence intervals will be generated using a mixed-effects log-binomial regression model, adjusting for the minimisation variables listed in section 8 and the patient's baseline results. All minimisation variables will be treated as fixed effects. All models will also adjust for site as a random effect to allow for the cluster design. A z-test will be used to test the statistical significance (p-value produced) of the estimated intervention group parameter generated from the maximum likelihood estimates.

Sensitivity analyses will be performed at a patient level based on learners who are adherent to domains of the educational intervention, as a whole and according to the domains separately (defined according to pre and post-testing, a minimum time using the online platform, appropriate required reading links clicked, interaction with the National Trainer, and completion of the commitment to change plan as assessed by the PI). An additional sensitivity analysis will be performed using a time-window around the follow-up assessment.

## 10.4 Other outcomes

The secondary outcomes for the proportion of guidelines attained and the proportion of patients treated with oral anticoagulants will be analysed using the same methods as described above for the co-primary outcomes. The Integrated AF management and EQ-5D-5L questionnaire responses will be converted to scores and analysed using a mixed linear regression model, adjusting for the intervention group, the score at baseline, and the minimisation variables listed in section 8 (site will also be included as a random effects variable). Mean differences between the two groups will be presented along with 95% confidence intervals. A z-test will be used to test the significance of the estimated intervention group parameter. The integrated AF management score will additionally be analysed using a mixed effect ordinal regression model adjusting for the intervention group, the score at baseline, and the minimisation variables listed in section 8 (site will also be included as a random effects variable). Odds ratios between the two groups will be presented along with 95% confidence intervals. A z-test will be used to test the statistical significance (p-value produced) of the estimated intervention group parameter generated from the maximum likelihood estimates.

Analysis of the major clinical events will be undertaken using time-to-event methodology (i.e. Kaplan-Meier and Cox proportional hazards model) where appropriate, along with a descriptive table of the events.

Process outcomes will be summarised for the intervention arm only using appropriate summary statistics, overall and by country.

DocuSign Envelope ID: 1B8B2092-5DB2-4866-899B-414B5B6B56FC

# 11. Trial Oversight

## 11.1 Trial Steering Committee

The role of the Trial Steering Committee (TSC) is to provide overall supervision of the trial. The TSC members are listed on page 4 and consist of the Chief Investigators, stroke and rhythm control leads, the six NCs, and representatives from the trials unit and coordinating centre. The TSC will monitor conduct of the trial, advise on scientific credibility and act, as appropriate, upon the recommendations of the Data Monitoring Committee. Monitoring by the TSC will include the percentage of healthcare practitioners at each centre who see AF patients on a daily basis and regularly participate in AF-specific education (e.g. ESC/EHRA courses/congresses).

The TSC and NCs will intercede if the proportion of these AF specialists exceed the trial monitoring plan by adapting future enrolment at sites. An interim analysis of actual between-cluster variability and intracluster correlation will be provided to the TSC at the mid-point of the target number of centres randomised. This is to assess whether the planned sample size is adequate. Any subsequent changes to the sample size will follow rules to preserve overall statistical significance of the trial.

## 11.2 Strategic Oversight Committee

The Strategic Oversight Committee (SOC) is an advisory board comprised of the leading stakeholders in STEEER-AF, including representation from the ESC Board, EHRA, ESC Education and the ESC Council on Stroke (members are listed on page 5). It also includes non-voting representation from the EURObservational Research Programme, the ESC and the TSC. The SOC will use its considerable experience to guide the TSC and also provide feedback to their constituent management boards and external stakeholders.

## 11.3 Data Monitoring Committee

An independent Data Monitoring Committee (DMC) will be established to oversee the safety of patients and healthcare professionals taking part in the trial. PIs will be able to communicate any safety issues directly to the DMC, or through their NC. The DMC will consist of the Chair, two experienced clinicians in the field of AF, and an independent statistician (see page 5). The DMC will meet prior to the trial opening to enrolment, and then annually, or according to a timetable agreed by the DMC prior to trial commencement. Meetings will consist of teleconferences or in-person sessions as required. The DMC will operate in accordance with a trial-specific DMC charter to be approved at the first meeting, based on the DAMOCLES recommendations.[27] Data analyses will be supplied in confidence to the DMC, which will be asked to give advice on the credibility of the study and validity of results. The DMC Chair will formally advise the TSC on recommendations of the DMC after each session, or as required.

Due to the current state of equipoise for the educational intervention (and no direct intervention to patients), no stopping rules are anticipated.

# 12. Consent

## 12.1 Cluster level

Cluster-level consent for the centre to be randomised to either the ESC educational programme or no additional ESC education will be sought via email from the department head for the relevant centre by the PI. Each PI will receive specific instruction from the NC, and will be expected to disseminate and discuss this information with Investigators at their centre.

This will form the Cluster Representation Mechanism (CRM)[28] for STEEER-AF, with each PI confirming in writing to their PI that the CRM considers the cluster's participation in the trial to be in the interests of the cluster as a whole, and in the interests of each member of the cluster (healthcare professionals and patients).

## 12.2 Patient level

Patients will be asked individually to consent to the collection of patient-level data after being provided a patient-information leaflet. The precise form of this consent (written, verbal or other) will depend on the requirements in each individual country, and stipulated in the relevant Ethical Committee documentation. Patients who do not provide this consent will not have any data collected. Patients enrolled in STEEER-AF are free to withdraw at any time, and this will not affect their ongoing clinical management. Data collected up to that time-point may still be used in analysis.

# 13. Ethics and Regulatory Requirements

## 13.1 Ethical principles

The ethical case for carrying out this trial is based on a state of equipoise, in that there is genuine uncertainty as to whether educational programmes targeted to physicians can improve patient-level outcomes in AF. Further, although numerous educational materials are available for physicians, these are (1) not currently focused to patient outcomes, (2) frequently ad-hoc or on a hospital-to-hospital basis, (3) of variable quality and (4) usually designed by industry-sponsored or other biased groups. STEEER-AF will provide unique data on the effectiveness of educating healthcare professionals for patient benefit, with a programme developed by clinical investigators and sponsored by a professional medical organisation.

DocuSign Envelope ID: 1B8B2092-5DB2-4866-899B-414B5B6B56FC

## 13.2 Ethical Approval

The trial will be performed in accordance with the recommendations guiding physicians in biomedical research involving human participants, adopted by the 18th World Medical Association General Assembly, Finland, June 1964, amended at the 48th World Medical Association General Assembly, Republic of South Africa, October 1996 (the Declaration of Helsinki, 1964.

The trial will be conducted in accordance with research governance frameworks in the relevant countries and the EU Clinical Trials Directive and Guidelines for Good Clinical Practice (GCP). The protocol will be submitted to and approved by the relevant Research Ethics Committees in each country, or at the individual participating institutions depending on local rules and regulations.

Before any participants are enrolled into the trial, the PI, supported by the Contract Research Organisation (CRO), is required to obtain all local approvals.

It is the responsibility of the PI to ensure that all subsequent amendments gain the necessary local approval. This does not affect the individual clinicians' responsibility to take immediate action if thought necessary to protect the health and interest of individual patients.

## 13.3 Confidentiality and Data Protection

Personal data recorded on all documents will be regarded as strictly confidential and will be handled and stored in accordance with local, EU (ie. GDPR etc.) and UK regulations.

Participants will be identified using their unique trial identification number. The CRO, coordinating centre and statistical unit will maintain the confidentiality of all participants' data and will not disclose information by which participants may be identified to any third party, other than those directly involved in the treatment of the participant and organisations for which the participant has given explicit consent for data transfer (e.g. competent local authority). Data will be archived, in accordance with local regulations.

## 13.4 Insurance and Indemnity

All issues concerning insurance, indemnity and the need for such aspects are the responsibility of the sponsor according to individual national requirements. See Section 6.6 for the benefit-risk assessment.

With respect to the conduct of the trial at each clinical site and other clinical care of the patient, responsibility for the care of any patients remains with the local health organisation responsible for that site and is therefore indemnified through local processes.

### 13.5 Statement of Compliance

The STEEER-AF trial will be conducted in compliance with the approved protocol, UK and EU GCP, and all applicable regulatory requirements.

## 14. Investigators, Disclosures and Dissemination

### 14.1 Investigators and financial disclosures

All persons working on the trial (TSC, DMC, SOC, trainers, PIs and Investigators) will be expected to disclose all relevant financial disclosures.

### 14.2 Dissemination and publication plan

Upon receipt of last patient data for 6-9 month follow-up, members of the TSC will form a Trial Publication Committee to oversee relevant dissemination of results. Named authors on publications must satisfy the International Committee of Medical Journal Editors (ICMJE) criteria for authorship (contribute to drafting of the article or revision for important intellectual content, provide timely approval of the final version to be published, and supply detailed statements on any potential conflict of interest or financial relationships). Members of the group who do not fulfil ICMJE criteria for authorship will be listed in the article appendix. Final results will be disseminated by ESC/EHRA throughout its network of cardiologists and allied health professionals.

### 14.3 Protocol and publication adherence

This trial protocol has been constructed in accordance with SPIRIT recommendations[29], and will be registered with the European Union Drug Regulating Authorities Clinical Trials Office (EudraCT) and clinicaltrials.gov. Reporting of the trial will follow guidance by CONSORT (specifically in relation to cluster trials).[30]

DocuSign Envelope ID: 1B8B2092-5DB2-4866-899B-414B5B6B56FC

# 15. References


**1.** P Kirchhof, S Benussi, D Kotecha, A Ahlsson, D Atar, B Casadei, . . . P Vardas. 2016 ESC Guidelines for the management of atrial fibrillation developed in collaboration with EACTS. *Eur Heart J.* 2016;37:2893-962.

**2.** DA Lane, F Skjoth, GYH Lip, TB Larsen, D Kotecha. Temporal Trends in Incidence, Prevalence, and Mortality of Atrial Fibrillation in Primary Care. *J Am Heart Assoc.* 2017;6:e005155.

**3.** D Kotecha, G Breithardt, AJ Camm, GYH Lip, U Schotten, A Ahlsson, . . . P Kirchhof. Integrating new approaches to atrial fibrillation management: the 6th AFNET/EHRA Consensus Conference. *Europace.* 2018;20:395-407.

**4.** R Nieuwlaat, SB Olsson, GY Lip, AJ Camm, G Breithardt, A Capucci, . . . HJ Crijns. Guideline-adherent antithrombotic treatment is associated with improved outcomes compared with undertreatment in high-risk patients with atrial fibrillation. The Euro Heart Survey on Atrial Fibrillation. *Am Heart J.* 2007;153:1006-12.

**5.** GY Lip, C Laroche, MI Popescu, LH Rasmussen, L Vitali-Serdoz, GA Dan, . . . G Boriani. Improved outcomes with European Society of Cardiology guideline-adherent antithrombotic treatment in high-risk patients with atrial fibrillation: a report from the EORP-AF General Pilot Registry. *Europace.* 2015;17:1777-86.

**6.** M Proietti, A Nobili, V Raparelli, L Napoleone, PM Mannucci, GY Lip. Adherence to antithrombotic therapy guidelines improves mortality among elderly patients with atrial fibrillation: insights from the REPOSI study. *Clin Res Cardiol.* 2016;105:912-20.

**7.** F Peters-Klimm, I Natanzon, T Muller-Tasch, S Ludt, C Nikendei, N Lossnitzer, . . . J Junger. Barriers to guideline implementation and educational needs of general practitioners regarding heart failure: a qualitative study. *GMS Z Med Ausbild.* 2012;29:Doc46.

**8.** F Fischer, K Lange, K Klose, W Greiner, A Kraemer. Barriers and Strategies in Guideline Implementation-A Scoping Review. *Healthcare (Basel).* 2016;4:36.

**9.** TA Glauser, J Barnes, H Nevins, W Cerenzia. The Educational Needs of Clinicians Regarding Anticoagulation Therapy for Prevention of Thromboembolism and Stroke in Patients With Atrial Fibrillation. *Am J Med Qual.* 2016;31:38-46.

**10.** B Freedman, TS Potpara, GY Lip. Stroke prevention in atrial fibrillation. *Lancet.* 2016;388:806-17.

**11.** R Nieuwlaat, A Capucci, AJ Camm, SB Olsson, D Andresen, DW Davies, . . . HJ Crijns. Atrial fibrillation management: a prospective survey in ESC member countries: the Euro Heart Survey on Atrial Fibrillation. *Eur Heart J.* 2005;26:2422-34.

**12.** GY Lip, C Laroche, G Boriani, GA Dan, M Santini, Z Kalarus, . . . L Tavazzi. Regional differences in presentation and treatment of patients with atrial fibrillation in Europe: a report from the EURObservational Research Programme Atrial Fibrillation (EORP-AF) Pilot General Registry. *Europace.* 2015;17:194-206.

**13.** JM Hendriks, R de Wit, HJ Crijns, HJ Vrijhoef, MH Prins, R Pisters, . . . RG Tieleman. Nurse-led care vs. usual care for patients with atrial fibrillation: results of a randomized trial of integrated chronic care vs. routine clinical care in ambulatory patients with atrial fibrillation. *Eur Heart J.* 2012;33:2692-9.

**14.** MV Huisman, KJ Rothman, M Paquette, C Teutsch, HC Diener, SJ Dubner, . . . GY Lip. The Changing Landscape for Stroke Prevention in AF: Findings From the GLORIA-AF Registry Phase 2. *J Am Coll Cardiol.* 2017;69:777-85.

**15.** P Kirchhof, B Ammentorp, H Darius, R De Caterina, JY Le Heuzey, RJ Schilling, . . . JL Zamorano. Management of atrial fibrillation in seven European countries after the publication of the 2010 ESC Guidelines on atrial fibrillation: primary results of the PREvention oF thromboemolic events--European Registry in Atrial Fibrillation (PREFER in AF). *Europace.* 2014;16:6-14.

**16.** D Kotecha, JJ Bax, C Carrera, B Casadei, B Merkely, SD Anker, . . . P Kirchhof. Roadmap for cardiovascular education across the European Society of Cardiology: inspiring better knowledge and skills, now and for the future. *Eur Heart J.* 2019;40:1728-38.

**17.** DL Packer, DB Mark, RA Robb, KH Monahan, TD Bahnson, JE Poole, . . . KL Lee. Effect of Catheter Ablation vs Antiarrhythmic Drug Therapy on Mortality, Stroke, Bleeding, and Cardiac Arrest Among Patients With Atrial Fibrillation: The CABANA Randomized Clinical Trial. *Jama.* 2019;321:1261-74.

DocuSign Envelope ID: 1B8B2092-5DB2-4866-899B-414B5B6B56FC

**18.** D Kotecha, WWL Chua, L Fabritz, J Hendriks, B Casadei, U Schotten, . . . European Heart Rhythm Association. European Society of Cardiology smartphone and tablet applications for patients with atrial fibrillation and their health care providers. *Europace.* 2018;20:225-33.

**19.** H Heidbuchel, N Dagres, M Antz, KH Kuck, P Lazure, S Murray, . . . A Vahanian. Major knowledge gaps and system barriers to guideline implementation among European physicians treating patients with atrial fibrillation: a European Society of Cardiology international educational needs assessment. *Europace.* 2018;20:1919-28.

**20.** D Vinereanu, RD Lopes, MC Bahit, D Xavier, J Jiang, HR Al-Khalidi, . . . CB Granger. A multifaceted intervention to improve treatment with oral anticoagulants in atrial fibrillation (IMPACT-AF): an international, cluster-randomised trial. *Lancet.* 2017;390:1737-46.

**21.** European Society of Cardiology. ESC Strategic Plan 2016-2020.https://www.escardio.org/The-ESC/About/esc-strategic-plan-2016-2020 [Accessed 20 December 2019].

**22.** DG Altman. *Practical statistics for medical research.* Boca Raton, USA: Chapman & Hall/CRC; 1999.

**23.** RJ Hayes, LH Moulton. *Cluster randomised trials.* Second edition. ed. Boca Raton: CRC Press; 2017.

**24.** GY Lip, C Laroche, PM Ioachim, LH Rasmussen, L Vitali-Serdoz, L Petrescu, . . . G Boriani. Prognosis and treatment of atrial fibrillation patients by European cardiologists: one year follow-up of the EURObservational Research Programme-Atrial Fibrillation General Registry Pilot Phase (EORP-AF Pilot registry). *Eur Heart J.* 2014;35:3365-76.

**25.** MK Campbell, PM Fayers, JM Grimshaw. Determinants of the intracluster correlation coefficient in cluster randomized trials: the case of implementation research. *Clin Trials.* 2005;2:99-107.

**26.** RB Schnabel, L Pecen, FM Ojeda, M Lucerna, N Rzayeva, S Blankenberg, . . . P Kirchhof. Gender differences in clinical presentation and 1-year outcomes in atrial fibrillation. *Heart.* 2017;103:1024-30.

**27.** DAMOCLES Study Group. A proposed charter for clinical trial data monitoring committees: helping them to do their job well. *Lancet.* 2005;365:711-22.

**28.** Medical Research Council. Cluster randomised trials: Methodological and ethical considerations. London: MRC; 2002.

**29.** AW Chan, JM Tetzlaff, PC Gotzsche, DG Altman, H Mann, JA Berlin, . . . D Moher. SPIRIT 2013 explanation and elaboration: guidance for protocols of clinical trials. *BMJ.* 2013;346:e7586.

**30.** MK Campbell, G Piaggio, DR Elbourne, DG Altman. Consort 2010 statement: extension to cluster randomised trials. *BMJ.* 2012;345:e5661.

DocuSign Envelope ID: 1B8B2092-5DB2-4866-899B-414B5B6B56FC

# Appendix 1

Extract of class I and III guideline recommendations from the 2016 ESC AF Guidelines

| Recommendations on stroke prevention | Class | Level |
|---|---|---|
| The $CHA_2DS_2$-VASc score is recommended for stroke risk prediction in patients with AF | I | A |
| Oral anticoagulation therapy to prevent thromboembolism is recommended for all male AF patients with a $CHA_2DS_2$-VASc score of 2 or more | I | A |
| Oral anticoagulation therapy to prevent thromboembolism is recommended in all female AF patients with a $CHA_2DS_2$-VASc score of 3 or more | I | A |
| Vitamin K antagonist therapy (INR 2.0–3.0 or higher) is recommended for stroke prevention in AF patients with moderate-to-severe mitral stenosis or mechanical heart valves | I | B |
| When oral anticoagulation is initiated in a patient with AF who is eligible for a NOAC (apixaban, dabigatran, edoxaban, or rivaroxaban), a NOAC is recommended in preference to a Vitamin K antagonist | I | A |
| When patients are treated with a vitamin K antagonist, time in therapeutic range (TTR) should be kept as high as possible and closely monitored | I | A |
| Combinations of oral anticoagulants and platelet inhibitors increase bleeding risk and should be avoided in AF patients without another indication for platelet inhibition | III (harm) | B |
| In male or female AF patients without additional stroke risk factors, anticoagulant or antiplatelet therapy is not recommended for stroke prevention | III (harm) | B |
| Antiplatelet monotherapy is not recommended for stroke prevention in AF patients, regardless of stroke risk | III (harm) | A |
| NOACs (apixaban, dabigatran, edoxaban, and rivaroxaban) are not recommended in patients with mechanical heart valves (Level of evidence B) or moderate-to-severe mitral stenosis (Level of evidence C) | III (harm) | B/C |
| After surgical occlusion or exclusion of the LAA, it is recommended to continue anticoagulation in at-risk patients with AF for stroke prevention | I | B |
| **Recommendations for secondary stroke prevention** | | |
| Anticoagulation with heparin or low-molecular-weight heparin immediately after ischaemic stroke is not recommended in AF patients | III (harm) | A |
| Systemic thrombolysis with a recombinant tissue plasminogen activator is not recommended if the INR is above 1.7 (or, for patients on dabigatran, if activated partial thromboplastin time is outside the normal range) | III (harm) | C |
| After TIA or stroke, combination therapy of OAC and an antiplatelet is not recommended | III (harm) | B |

DocuSign Envelope ID: 1B8B2092-5DB2-4866-899B-414B5B6B56FC

# Appendix 2

Extract of class I and III guideline recommendations from the 2016 ESC AF Guidelines

| General recommendations on rhythm control | Class | Level |
|---|---|---|
| Use of the modified EHRA symptom scale is recommended in clinical practice and research studies to quantify AF-related symptoms | I | C |
| Rhythm control therapy is indicated for symptom improvement in patients with AF | I | B |
| **Cardioversion of AF** | | |
| Electrical cardioversion of AF is recommended in patients with acute haemodynamic instability to acutely restore cardiac output | I | B |
| Cardioversion of AF (either electrical or pharmacological) is recommended in symptomatic patients with persistent or long-standing persistent AF as part of rhythm control therapy | I | B |
| In patients with no history of ischaemic or structural heart disease, flecainide, propafenone, or vernakalant are recommended for pharmacological cardioversion of new-onset AF | I | A |
| In patients with ischaemic and/or structural heart disease, amiodarone is recommended for cardioversion of AF | I | A |
| **Stroke prevention in patients designated for cardioversion of AF** | | |
| For cardioversion of AF/atrial flutter, effective anticoagulation is recommended for a minimum of 3 weeks before cardioversion | I | B |
| Transoesophageal echocardiography (TOE) is recommended to exclude cardiac thrombus, as an alternative to preprocedural anticoagulation when early cardioversion is planned | I | B |
| In patients at risk for stroke (e.g. presence of $CHA_2DS_2$-VASc factors), anticoagulant therapy should be continued long-term after cardioversion according to the long-term anticoagulation recommendations, irrespective of the method of cardioversion or the apparent maintenance of sinus rhythm. In patients without stroke risk factors, anticoagulation is recommended for 4 weeks after cardioversion | I | B |
| In patients where thrombus is identified on TOE, effective anticoagulation is recommended for at least 3 weeks | I | C |
| **Antiarrhythmic drugs for long-term maintenance of sinus rhythm/prevention of recurrent AF** | | |
| The choice of antiarrhythmic drug needs to be carefully evaluated, taking into account the presence of comorbidities, cardiovascular risk and potential for serious proarrhythmia, extracardiac toxic effects, patient preferences, and symptom burden | I | A |
| Dronedarone, flecainide, propafenone, or sotalol are recommended for prevention of recurrent symptomatic AF in patients with normal left ventricular function and without pathological left ventricular hypertrophy. | I | A |

DocuSign Envelope ID: 1B8B2092-5DB2-4866-899B-414B5B6B56FC

| | | |
|---|---|---|
| Dronedarone is recommended for prevention of recurrent symptomatic AF in patients with stable coronary artery disease, and without heart failure | I | A |
| Amiodarone is recommended for prevention of recurrent symptomatic AF in patients with heart failure | I | B |
| Antiarrhythmic drug therapy is not recommended in patients with prolonged QT interval (> 0.5 s) or with significant sinoatrial node disease or atrioventricular node dysfunction who do not have a functioning permanent pacemaker | III (harm) | C |
| **Antiarrhythmic effects of non-antiarrhythmic drugs** | | |
| ARBs or ACE inhibitors are not recommended for the secondary prevention of paroxysmal AF in patients with little or no underlying heart disease. | III (no benefit) | B |
| **Catheter ablation of AF and AF surgery** | | |
| Catheter ablation of symptomatic paroxysmal AF is recommended to improve AF symptoms in patients who have symptomatic recurrences of AF on antiarrhythmic drug therapy (amiodarone, dronedarone, flecainide, propafenone, sotalol) and who prefer further rhythm control therapy, when performed by an electrophysiologist who has received appropriate training and is performing the procedure in an experienced centre | I | A |
| **Wolff-Parkinson-White (WPW) syndrome** | | |
| Catheter ablation of the accessory pathway in WPW patients with AF and rapid conduction over the accessory pathway is recommended to prevent sudden cardiac death | I | B |
| Catheter ablation of the accessory pathway is recommended without delay in WPW patients who survive sudden cardiac death | I | C |
| **Hypertrophic cardiomyopathy** | | |
| Lifelong oral anticoagulation to prevent stroke is recommended in hypertrophic cardiomyopathy patients who develop AF | I | B |
| Restoration of sinus rhythm by electrical or pharmacological cardioversion to improve symptoms is recommended in hypertrophic cardiomyopathy patients with symptomatic new-onset AF | I | B |

# Stroke prevention and rhythm control Therapy: Evaluation of an Educational Programme of the European society of cardiology in a Cluster-Randomised trial in patients with Atrial Fibrillation

# The STEEER-AF Trial

EudraCT number: 2020-000792-20
ClinicalTrials.org: NCT04396418

# Statistical Analysis Plan

| SAP Version Number | Protocol Version Number |
|---|---|
| 2.0 | 1.0 |

| | | | | | |
|---|---|---|---|---|---|
| Name of Author: | Samir Mehta | Role: | Senior Statistician | Affiliation: | BCTU University of Birmingham |
| Signature of Author: | Samir Mehta (eSigned) | Date: | 03/05/2024 | | |
| Name of statistician: | Dr Yongzhong Sun | Role: | Trial Statistician | Affiliation: | BCTU University of Birmingham |
| Signature of statistician: | Yongzhong Sun (eSigned) | Date: | 17/05/2024 | | |
| Name of Reviewer: | Becky Woolley | Role: | Senior Statistician | Affiliation: | BCTU University of Birmingham |
| Signature of reviewer: | Becky Woolley (eSigned) | Date: | 16/05/2024 | | |
| Name of Chief Investigator: | Prof. Dipak Kotecha | Role: | Chief Investigator | Affiliation: | University Hospitals Birmingham NHS Foundation Trust University of Birmingham |
| Signature of Chief Investigator: | Dipak Kotecha (eSigned) | Date: | 15/05/2024 | | |
| Name of Chief Investigator: | Prof. Isabelle van Gelder | Role: | Co-Chief Investigator | Affiliation: | University Medical Centre Groningen, Netherlands |
| Signature of Chief Investigator: | Isabelle van Gelder (eSigned) | Date: | 15/05/2024 | | |
| **This Statistical Analysis Plan has been reviewed and approved by:** | | | | | |
| Name of Approver: | Samir Mehta | Role: | Senior Statistician | Affiliation: | BCTU University of Birmingham |
| Signature of Approver: | Samir Mehta (eSigned) | Date: | 17/05/2024 | | |

## Statistical Analysis Plan (SAP) Amendments

| SAP version number | SAP section number | Description of and reason for change | Timing of change with respect to interim analysis/ final analysis/ database lock | Blind Reviewer | |
|---|---|---|---|---|---|
| 2.0 | 9.5 | Update to the statistical analysis method for co-primary outcome as both are binary outcome data and originally were planned to be analysed using a mixed effects binomial model to estimate the RR and RD. However since the writing of the original version 1.0 of the SAP, there have been many recent paper recommending not to use models with non-canonical link and so in line with these recommendations, the analysis methods have been updated. And so now a mixed effects logistic model will be fitted and marginal standardisation approach will be used to estimate the RR and RD. | Before final analysis and data lock | Name: | Becky Woolley |
| | | | | Signature: | esign – B. Woolley |
| | | | | Date: | 16/05/2024 |
| 2.0 | 9.9 | Update to the subgroup analysis section to match the new analysis methods mentioned in section 9.5 as well as adding the original analysis method of using mixed effects binomial regression model for co-primary outcome as sensitivity analysis. | Before final analysis and data lock | Name: | Becky Woolley |
| | | | | Signature: | esign – B. Woolley |
| | | | | Date: | 16/05/2024 |
| 2.0 | 9.10 | Update to the missing data imputation approach as previously we stated that a best case and worst case imputation method will be adopted. However since the writing of the original version 1.0 of the SAP, there has been a better method to impute binary outcome missing data which explores the possibility that missing responses are 'missing not at random' (MNAR) using a tipping point approach. And so this approach is more robust compared to previous one hence the change. | Before final analysis and data lock | Name: | Becky Woolley |
| | | | | Signature: | esign – B. Woolley |
| | | | | Date: | 16/05/2024 |
| 2.0 | 13 | Addition of references. | Before final analysis and data lock | Name: | Becky Woolley |
| | | | | Signature: | esign – B. Woolley |
| | | | | Date: | 16/05/2024 |

| Abbreviations & Definitions | |
|---|---|
| **Abbreviation / Acronym** | **Meaning** |
| BCTU | Birmingham Clinical Trials Unit |
| CONSORT | Consolidated Standards of Reporting Trials |
| DMC | Data Monitoring Committee |
| ISRCTN | International Standard Randomised Controlled Trial Number |
| ITT | Intention to Treat |
| SAE | Serious Adverse Event |
| SAP | Statistical Analysis Plan |
| SUSAR | Suspected Unexpected Serious Adverse Reaction |
| TSC | Trial Steering Committee |
| AF | Atrial fibrillation |
| EHRA | European Heart Rhythm Association |
| ESC | European Society of Cardiology |
| NOAC | Non-Vitamin K antagonist oral anticoagulant |
| | |
| **Term** | **Definition** |
| International Standard Randomised Controlled Trial Number | A clinical trial registry |
| Protocol | Document that details the rationale, objectives, design, methodology and statistical considerations of the study |
| Randomisation | The process of assigning trial subjects to intervention or control groups using an element of chance to determine the assignments in order to reduce bias. |
| Statistical Analysis Plan | Pre-specified statistical methodology documented for the trial, either in the protocol or in a separate document. |
| | |

# TABLE OF CONTENTS

1. Introduction ..... 7
2. Background and rationale ..... 7
3. Trial objectives ..... 8
4. Trial methods ..... 8
4.1. Trial design ..... 8
4.2. Trial interventions ..... 8
4.3. Co-Primary outcome measures ..... 9
4.4. Secondary outcome measures ..... 9
4.5. Timing of outcome assessments ..... 9
4.6. Randomisation ..... 10
4.7. Sample size ..... 11
4.8. Framework ..... 11
4.9. Interim analyses and stopping guidance ..... 11
4.10. Internal Pilot Progression Rules ..... 12
4.11. Timing of final analysis ..... 12
4.12. Timing of other analyses ..... 12
4.13. Trial comparisons ..... 12
5. Statistical Principles ..... 12
5.1. Confidence intervals and p-values ..... 12
5.2. Adjustments for multiplicity ..... 12
5.3. Analysis populations ..... 12
5.4. Definition of adherence ..... 13
5.5. Handing protocol deviations ..... 13
5.6. Unblinding ..... 13
6. Trial population ..... 13
6.1. Recruitment ..... 13
6.2. Baseline characteristics ..... 14
7. Intervention ..... 14
7.1. Description of the intervention ..... 14
7.2. Adherence to allocated intervention ..... 15
8. Protocol deviations ..... 15
9. Analysis methods ..... 15
9.1. Covariate adjustment ..... 15
9.2. Distributional assumptions and outlying responses ..... 16
9.3. Handling missing data ..... 16
9.4. Data manipulations ..... 16
9.5. Analysis methods – primary outcome(s) ..... 17
9.6. Analysis methods – secondary outcomes ..... 18
9.7. Analysis methods – exploratory outcomes and analyses ..... 19
9.8. Safety data ..... 19
9.9. Planned subgroup analyses ..... 19
9.10. Sensitivity analyses ..... 20
10. Analysis of sub-randomisations ..... 21
11. Health economic analysis ..... 22
12. Statistical software ..... 22
13. References ..... 22
Appendix A: Deviations from SAP ..... 24
Appendix B: Trial schema ..... 24
Appendix C: Schedule of assessments ..... 25
Appendix D: Template report ..... 25

## 1. Introduction

This document is the Statistical Analysis Plan (SAP) for the STEEER-AF trial, and should be read in conjunction with the current trial protocol. This SAP details the proposed analyses and presentation of the data for the main paper(s) reporting the results for the STEEER-AF trial.

The results reported in these papers will follow the strategy set out here. Subsequent analyses of a more exploratory nature will not be bound by this strategy, though they are expected to follow the broad principles laid down here. The principles are not intended to curtail exploratory analysis (e.g. to decide cut-points for categorisation of continuous variables), nor to prohibit accepted practices (e.g. transformation of data prior to analysis), but they are intended to establish rules that will be followed, as closely as possible, when analysing and reporting data.

Any deviations from this SAP will be described and justified in the final report or publication of the trial (using a table as shown in Appendix A). The analysis will be carried out by an appropriately qualified statistician, who should ensure integrity of the data during their data cleaning processes.

## 2. Background and rationale

The background and rationale for the trial are outlined in detail in the protocol.

In brief, atrial fibrillation (AF) is a heart condition that causes an irregular and often abnormally fast heart rate. It is common, will continue to become more prevalent in the years to come, and is associated with considerable burden to both patients and healthcare services. Healthcare professionals that manage patients with AF are faced with numerous challenges to provide care that improves the outcomes of patients with AF, including therapies for stroke prevention and rhythm control. These two facets of AF management are complex, relying on integration of patient-level factors, locally-available treatment strategies and appropriate decision-making by physicians. Hence, the burden of AF extends to the education and knowledge-base of those treating patients with AF.

The European Society of Cardiology (ESC) provides practice guidelines for a number of cardiovascular conditions, including AF.[1-2] Guideline-adherent care is defined as the implementation of guideline recommendations to individual patients. Guideline-adherent treatment has been shown to improve the outcomes of patients with AF, including lower rates of mortality, incident stroke and major bleeding.

The challenge for healthcare services is that guidelines are often not applied, applied partially, or even incorrectly, with the education and training of healthcare workers identified as major barriers for guideline implementation.

## 3. Trial objectives

The primary objective is to determine whether a comprehensive and structured educational programme for healthcare professionals treating patients with AF, compared to no added education, will improve guideline-adherent provision of patient-level treatments relating to stroke prevention and rhythm control.

Secondary objectives are to determine whether a comprehensive and structured educational programme for healthcare professionals treating patients with AF, compared to no added education, will improve the proportion of guidelines implemented at patient-level and the provision of integrated care, lead to benefit in patient-reported quality of life and reduce major adverse clinical outcomes.

## 4. Trial methods

### 4.1. Trial design

STEEER-AF is a pragmatic, international, multi-centre, parallel group, two-arm, unblinded, cluster-randomised controlled trial.

Patients will be recruited from hospitals and health centres in six European countries (France, Germany, Italy, Poland, Spain and the United Kingdom). Individual hospitals and health centres in these six countries will be the clusters (defined as a centre recruiting patients).

### 4.2. Trial interventions

STEEER-AF is a cluster-randomised control trial with the clusters (i.e. centre recruiting patients) being randomised to educational intervention group or control group.

Intervention group:

- The intervention is a comprehensive and structured educational programme designed by stakeholders from the ESC Education Committee, EHRA and ESC Council on Stroke for healthcare professionals treating patients with AF.
- It is a 16 week education intervention programme targeted towards stroke prevention and rhythm control, previously identified as key educational gaps.

Control group:

The centres randomised to the control group will not receive the educational intervention, but will participate in data collection of patient level data at baseline, follow-up at 6-9 months post-randomisation and remote clinical events.

## 4.3. Co-Primary outcome measures

The Co-primary outcomes are:

- Guideline-adherence for stroke prevention therapy, based on class I and III recommendations of the ESC 2016 AF Guidelines
- Guideline-adherence for rhythm control therapy, based on the class I and III recommendations of the ESC 2016 AF Guidelines

## 4.4. Secondary outcome measures

The secondary outcomes are as follows:

- Proportion of guidelines adhered to for stroke prevention relevant to that patient, based on class I and III recommendations of the ESC 2016 AF Guidelines
- Proportion of guidelines adhered to for rhythm control relevant to that patient, based on class I and III recommendations of the ESC 2016 AF Guidelines
- Proportion of eligible patients treated with oral anticoagulants, including both class I and II guideline indications (women with $CHA_2DS_2$-VASc score 2 or above; men with $CHA_2DS_2$-VASc score 1 or above)
- Proportion of patients treated with oral anticoagulants according to class I guideline indications (women with $CHA_2DS_2$-VASc score 3 or above; men with $CHA_2DS_2$-VASc score 2 or above)
- Integrated AF management score
- Patient-reported quality of life using the EQ-5D-5L for overall mean index value and visual analogue scale

## 4.5. Timing of outcome assessments

The schedule of trial procedures and outcome assessments are given in Appendix C.

### 4.6. Randomisation

Randomisation will be performed only after the centre has enrolled their allocation of patients and baseline data has been transferred to the Birmingham Clinical Trials Unit (BCTU).

The National PI and Chief Investigators will make a joint decision on the allocation number for that centre based on recruitment at other centres in that country (overall target 25 patients per centre). Prior to randomisation, eligibility will be checked at both cluster and patient-level.

Centres will be randomised in a 1:1 ratio to either educational intervention or usual care.

A minimisation algorithm will be used to ensure balance between the 2 groups over the following variables:

- Country
  - (UK, France, Italy, Spain, Poland, Germany)
- Cluster-specific mean for class I and III guideline adherence to stroke prevention at baseline in enrolled patients
  - ($<70$, $\geq 70\%$)
- Cluster-specific mean for class I and III guideline adherence to rhythm control at baseline in enrolled patients
  - ($<50$, $\geq 50\%$)

The allocation will be generated by the statistician at BCTU, with blinding to the identity of the centres. The statistician will inform the trial coordinating centre at the ESC, who will then notify the Centre PI and arrange for deployment of the educational programme to centres randomised to the intervention group. This local deployment will include generating personal login details for the nominated healthcare professional learners in that institution, and contact with the National Trainer responsible for that centre.

Due to the nature of the intervention, it is not possible to blind hospital or health centre staff to the randomised allocation. However, to minimise selection bias, centres will not be informed of their allocated intervention until they have recruited the required number of patients.

Further recruitment of patients into the study will not be undertaken once the centre has been made aware of their allocated group.

## 4.7. Sample size

Based on published surveys, 80% of control patients are expected to receive guideline-adherent care for stroke prevention.[3-4] A relative increase of 10% (i.e. absolute increase of 8% from 80% to 88%) is considered by the trial team as a clinically-relevant improvement in guideline adherence for stroke prevention therapy. A global educational trial for stroke prevention used an intracluster correlation coefficient (ICC) of 0.02.[5] As a conservative measure, our sample size calculations are based on a higher ICC of 0.04.[6] With a 5% two-sided alpha, cluster size of 25 patients, 70 clusters in total, 1750 patients and this ICC of 0.04, STEEER-AF will have a power of 85% for this outcome. This includes 10% loss to patient follow-up and a small amount of variation in cluster size (or loss of clusters), with an assumed coefficient of variation of cluster size of 0.20.

For the rhythm control co-primary outcome, estimates of the control group rate are 50%.[3, 7] With the same assumptions as above, the sample size would provide 85% power to detect an absolute increase in guideline-adherence for rhythm control from 50% to 61%.

## 4.8. Framework

The objective of the trial is to test the superiority of one intervention to another.

The null hypothesis is that there is no difference in the proportion of relevant guideline recommendations attained for stroke prevention or rhythm control during follow-up at patient-level, comparing the structured educational programme to no added education of healthcare professionals. The alternative hypothesis is that there is a difference between the groups.

## 4.9. Interim analyses and stopping guidance

An independent Data Monitoring Committee (DMC) will be established to oversee the safety of patients and healthcare professionals taking part in the trial. Centre PIs will be able to communicate any safety issues directly to the DMC, or through their National PI. The DMC will consist of the Chair, two experienced clinicians in the field of AF, and an independent statistician.

The DMC will meet prior to the trial opening to enrolment, and then annually, or according to a timetable agreed by the DMC prior to trial commencement. Meetings will consist of teleconferences or in-person sessions as required. The DMC will operate in accordance with a trial-specific DMC charter to be approved at the first meeting, based on the DAMOCLES recommendations.[8] Data analyses will be supplied in confidence to the DMC, which will be asked to give advice on the credibility of the study and validity of results. The DMC Chair will formally advise the TSC on recommendations of the DMC after each session, or as required. Due to the current state of equipoise for the educational intervention (and no direct intervention to patients), no stopping rules are anticipated.

## 4.10. Internal Pilot Progression Rules

Not applicable.

## 4.11. Timing of final analysis

The final analysis for the trial will occur after all of the patients have been randomised, their 6-9 month follow up has been completed and the corresponding outcome data has been entered onto the trial database and validated as being ready for analysis. This is provided that the trial has not been stopped early for any reason (e.g. DMC advice or funding body request).

## 4.12. Timing of other analyses

Further analysis will occur after the remote follow-up for clinical events at 18 months from randomisation. The analysis methods for this will be described in this SAP as part of the safety data section 9.8.

## 4.13. Trial comparisons

All references in this document to 'group' refer to intervention group or control group. The primary comparison groups will be composed of patients treated in centres receiving the intervention, versus patients in centres receiving control.

# 5. Statistical Principles

## 5.1. Confidence intervals and p-values

All estimates of differences between groups will be presented with two-sided 95% confidence intervals, unless otherwise stated. P-values will be reported from two-sided tests at the 5% significance level.

## 5.2. Adjustments for multiplicity

No correction for multiple testing will be made.

## 5.3. Analysis populations

All primary analyses (primary and secondary outcomes including safety outcomes) will be by intention-to-treat (ITT) principle, i.e. all clusters and patients will be analysed in the intervention group to which they were randomised, irrespective of protocol deviations.

### 5.4. Definition of adherence

Since this is a cluster randomised trial, adherence to allocated intervention will be monitored in the intervention arm only since the clusters randomised to the control group will not cross-over to the intervention group.

The following components of the intervention arm will be summarised using appropriate summary statistics:

1. Multiple choice questions following the learning objectives of each educational module included in the online intervention. The assessment will occur at the start of each learner's educational programme (pre-test) and are duplicated at the end of the 16-week educational intervention (post-test).
2. Time spent on the online platform for each learner.
3. Percentage of required reading links clicked by each learner.
4. In-person interaction of the learner with the National Trainer.
5. Commitment to change plan to assess local change in clinical practice.

### 5.5. Handing protocol deviations

A protocol deviation is defined as a failure to adhere to the protocol such as errors in applying the inclusion/exclusion criteria, the incorrect intervention being given, incorrect data being collected or measured, follow-up visits outside the visit window or missed follow-up visits. We will apply a strict definition of the ITT principle and will include all patients as per the ITT population described in section 5.3 in the analysis, in some form, regardless of deviation from the protocol.[9] This does not include those patients who have specifically withdrawn consent for the use of their data in the first instance; however these outcomes will be explored as per other missing responses.

### 5.6. Unblinding

Not applicable, STEEER-AF is an open-label cluster randomised trial.

## 6. Trial population

### 6.1. Recruitment

A flow diagram (as recommended by CONSORT[10]) will be produced to describe the patient flow through each stage of the trial. This will include information on the number (with reasons) of losses to follow-up (drop-outs and withdrawals) over the course of the trial.

## 6.2. Baseline characteristics

Categorical data will be summarised by number of patients, counts and percentages. Continuous data will be summarised by the number of patients, mean and standard deviation if deemed to be normally distributed or number of patients, median and interquartile range if data are skewed, and ranges if appropriate. Tests of statistical significance will not be undertaken, nor confidence intervals presented.[11]

# 7. Intervention

## 7.1. Description of the intervention

The intervention is a comprehensive and structured educational programme designed by stakeholders from the ESC Education Committee, EHRA and ESC Council on Stroke for healthcare professionals treating patients with AF. The programme is targeted towards stroke prevention and rhythm control, previously identified as key educational gaps.

It is a 16 week education intervention programme targeted towards stroke prevention and rhythm control, previously identified as key educational gaps. The educational programme will operate using a "train-the-trainer" approach, whereby trained trainers will organise a country-specific training programme according to pre-defined learning outcomes in modules developed by the Trial Steering Committee.

In brief, trainers in each country will be responsible for supervising and interacting online with Investigators in addition to face-to-face sessions (the Investigators are healthcare professionals who are involved in the care of AF patients at each centre). The educational programme will consist of 4 modules using a blended learning approach, each incorporating pre-reading, online resources, case based peer interaction, in-person education, and evaluation of course materials and attained knowledge using pre and post-testing.

Investigators at centres randomised to the intervention will be expected to devote 2-3 hours per week to the online components of the educational programme, with further clinically-relevant education according to the local needs of each learner. The programme will be submitted for accreditation, and patients will be able to claim a Continued Medical Education (CME) certificate after completion of the programme.

A key part of the intervention process is generation of a locally-derived 'commitment to change' plan which will encompass elements that encourage and help a broad range of staff in each centre to manage patients with AF better (i.e. indirect benefits from the educational programme). These plans will include participation from the PI, and are expected to upskill staff at the centre and provide long-lasting improvements to patient care.

A plan of the intervention is presented in Table below:

| Week | Educational intervention (total 16 week programme) |
|---|---|
| 1 | Consideration of local knowledge and needs, with quantitative pre-test for all learners |
| 2-6 | Blended learning experience, using a dedicated ESC platform for tailored learning, supported and moderated by the National Trainer. |
| 7 | Quantitative post-testing for all trainees. First draft of an individualised and local 'commitment to change' plan in discussion with the National Trainer and PI. |
| 8 | Face-to-face workshop with the National Trainer. Finalisation of 'commitment to change' plan. |
| 9-15 | Reinforcing activities and iterative feedback on actions to implement the 'commitment to change' plan. |
| 16 | Quantitative post-test for all trainees. |

## 7.2. Adherence to allocated intervention

Adherence to allocated intervention by the adherence categories stated in section 5.4 will be summarised using appropriate summary statistics.

# 8. Protocol deviations

Frequencies and percentages by allocation group will be produced for any protocol deviations.

# 9. Analysis methods

Intervention groups will be compared using mixed effects models, or a similar method, to adjust for all covariates as specified in section 9.1, where possible.

## 9.1. Covariate adjustment

Since this is a cluster randomised trial, the randomisation algorithm uses the cluster specific minimisation criteria. However, for the final analysis, we will be analysing individual patient data. Therefore, instead of adjusting for the cluster specific minimisation variables we will adjust for the individual patient specific criteria as fixed effects, i.e. whether each patient was guideline adherent (yes/no) for stroke prevention and rhythm control at randomisation as well as fixed effect parameter for the country from which the patient was recruited. Centre will be adjusted for in the model as a random effect parameter to allow for the cluster design.

In the first instance, intervention effects between groups for all outcomes will be adjusted for the patient specific variables mentioned above, country, and also for baseline score (where available).

If covariate adjustment is not possible (e.g. the model does not converge), covariates will be removed until the model converges, or unadjusted estimates will be produced, and it will be made clear in the final report.

## 9.2. Distributional assumptions and outlying responses

Distributional assumptions (e.g. normality of regression residuals for continuous outcomes) will be assessed visually prior to analysis; although in the first instance the proposed primary method of estimation in this analysis plan will be followed. If responses are considered to be particularly skewed and/or distributional assumptions violated, the impact of this will be examined through sensitivity analysis; this will consist of transformation of responses prior to analysis (e.g. log transformation) in the first instance.

## 9.3. Handling missing data

In the first instance, analysis will be completed on received data only with every effort made to follow-up patients to minimise any potential for bias. To examine the possible impact of missing data on the results, and to make sure we are complying with the intention-to-treat principle, sensitivity analyses will be performed on the co-primary outcome measure.[12] See section 9.10 for further details regarding sensitivity analyses.

## 9.4. Data manipulations

**Guideline adherence for co-primary outcomes**

The main data manipulations required for this trial all relate to the guideline adherence for the co-primary outcomes. However to maintain blinding of the investigators, this information cannot be included in the SAP. This is because if we were to add the details of this in the SAP, then this would contaminate the control arm as essentially the data manipulations would describe in detail what is necessary to achieve full guideline adherence for both co-primary outcomes.

Therefore to avoid this bias and prevent contamination of the control arm, it has been decided to describe this separately. A decision tree diagram will be produced for each of the co-primary outcomes based on the recommendations of the ESC 2016 and 2020 AF Guidelines. This will be used to map against the data collected in the database and CRF in order to create a statistical analysis program which will compute the guideline adherence as overall "Yes/No" for the co-primary outcomes. The program will also compute the proportion of guidelines that were adhered to for each patient.

Given the baseline guideline adherence is to be used as part of minimisation variables for randomisation, the algorithms for both co-primary outcomes will need to be completed and validated prior to randomisation of any centres.

**EQ-5D (5 level)**

The current NICE guidelines (updated October 2019) on the use of EQ-5D-5L scoring based on the most recent value set for England published by Devlin et al. 2018 was not to use this and instead to map the 5L data into 3L value set based on mapping function developed by van Hout et al. 2012. EQ-5D-5L have developed the crosswalk value sets for the 5L to 3L and so these values will be used for scoring: (https://euroqol.org/eq-5d-instruments/eq-5d-5l-about/valuation-standard-value-sets/crosswalk-index-value-calculator/).

For those patients that die prior to completing the EQ5D questionnaire, for the Index score a value of "0" will be imputed since for this questionnaire, a value of 0=death.

## 9.5. Analysis methods – primary outcome(s)

**The Co-primary outcomes are:**

- Guideline-adherence for stroke prevention therapy, based on class I and III recommendations of the ESC 2016 AF Guidelines
- Guideline-adherence for rhythm control therapy, based on the class I and III recommendations of the ESC 2016 AF Guidelines

Both outcomes are binary outcomes (yes/no) and so the number and percentage of patients that are guideline adherent for stroke prevention and rhythm control will be reported separately by allocation group.

Both outcomes will be analysed separately using a mixed effects logistic regression model. Model will be adjusted for the variables as specified in section 9.1. Risk ratios and risk differences will then be derived using marginal standardisation method[13], as in for example a CRT by Kirkwood et al[14].

Under this approach, the mean risk under the control condition and intervention condition is obtained. The risk ratio is then derived using the mean risk in intervention condition and by the mean risk in the control condition, with the calculation being performed on the log scale. The risk difference is derived as the mean risk in intervention condition minus the mean risk in the control condition. We will use the unconditional standard errors to obtain the confidence interval, as this allows for correlation amongst the observations.[15]

## 9.6. Analysis methods – secondary outcomes

- **Proportion of relevant guidelines adhered to for stroke prevention to that patient**
- **Proportion of relevant guidelines adhered to for rhythm control to that patient**
- **Proportion of eligible patients treated with oral anticoagulants including both class I and II guideline indications** (women with $CHA_2DS_2$-VASc score 2 or above; men with $CHA_2DS_2$-VASc score 1 or above)
- **Proportion of eligible patients treated with oral anticoagulants according to class I guideline indications** (women with $CHA_2DS_2$-VASc score 3 or above; men with $CHA_2DS_2$-VASc score 2 or above)

Each of these outcomes will also be converted to a percentage score and so these outcomes will be treated as continuous proportion data. Data for these four outcomes will be summarised separately by allocation group using mean and standard deviation along with minimum and maximum values. Since these outcomes are all proportions, the data will be bounded between the values [0, 1] and so a fractional regression model using a logit link will be fitted with cluster-robust standard errors. Marginal effects will be derived as mean differences in proportions between the groups and the associated 95% confidence intervals.

- **Integrated AF management approach (education, lifestyle support, self-management shared decision making, support tools, adherence and multidisciplinary management)**

The data for this outcome is a score out of 8 with higher scores indicating better outcomes. The score out of 8 will be converted as a percentage score and so outcome will be treated as continuous proportion data. Given this outcome is a proportion, the data will be bounded between the values [0, 1] and so again a fractional regression model using a logit link will be fitted with cluster-robust standard errors. Marginal effects will be derived as mean differences in proportions between the groups and the associated 95% confidence intervals.

- **Patient-reported quality of life using the EQ-5D-5L questionnaire**

The data for these outcomes are continuous and the computation for the index summary score is described in the data manipulations section 9.4. The visual analogue score (VAS) is obtained from a scale so this score does not need to be derived. The mean and standard deviation along with minimum and maximum values for index summary score and VAS score will be presented by allocation group.

An adjusted mean difference along with the corresponding 95% confidence interval will be estimated from a mixed effects linear regression model. Separate models will be fitted for the index summary score and the visual analogue score.

## 9.7. Analysis methods – exploratory outcomes and analyses

Any data that does not form a pre-specified outcome will be presented using simple summary statistics by intervention group (i.e. numbers and percentages for binary data and means (or medians) and standard deviations (or inter-quartile ranges) for continuous normal (or non-normal) data.

## 9.8. Safety data

STEEER-AF is directed at healthcare professionals, with cluster randomisation of additional education to these practitioners based on published best-practice guidelines. As no intervention is directed at patient-level, serious adverse event (SAE) reporting will not take place (from the patient perspective, STEEER-AF is an observational cohort design). However as part of the trial, there will be a remote follow-up period where data on major clinical events during the 18-month period after randomisation will be collected. Information on all-cause mortality, non-fatal stroke, transient ischaemic attack, pulmonary embolus, systemic embolic event, acute coronary syndrome, myocardial infarction, hospitalisation for heart failure, and/or major and clinically-relevant non-major bleeding (defined as requiring a hospital admission) will be collected. These data will be summarised by allocation group.

## 9.9. Planned subgroup analyses

Interpretation of subgroup analysis will be treated with caution (output will be treated as exploratory rather than definitive).[16]

<u>Analysis will be limited to the co- primary outcomes only, and for the following subgroups</u>:

- Country (UK, France, Italy, Spain, Poland, Germany)
- Baseline cluster-specific mean for class I and III guideline adherence to stroke prevention (<70, ≥70%)
- Baseline cluster-specific mean for class I and III guideline adherence to rhythm control (<50, ≥50%)
- Gender of patients (Male, Female) [excluding transgender]
- Age of patients (<65 years, ≥65 years)
- mEHRA score of patients at baseline (1/2a, 2b/3/4)
- $CHA_2DS_2$-VASc score of patients at baseline (0-3, 4 or more)
- Investigator type (Cardiology, non-cardiology)
- Gender of investigators (Male, Female)

The effects of these subgroups will be examined by including the relevant subgroup by treatment interaction term in the mixed effects logistic regression model for each separate subgroup analysis. Tests of heterogeneity will be presented along with subgroup specific relative risks and risk difference along with corresponding 95% confidence intervals.

## 9.10.Sensitivity analyses

Sensitivity analyses will be performed at a patient level, limited to the co-primary outcomes and will consist of:

- Learners who are adherent to all domains of the educational intervention, as a whole
- An analysis to assess the effect of missing responses for the co-primary outcomes only. This analysis will explore the possibility that missing responses are 'missing not at random' (MNAR) using a tipping point approach. In this analysis, for patients with missing outcome data, events will be added sequentially in each of the groups in turn to determine the point where the general conclusion changes. An assessment can then be made about whether the event rate in the missing responses between groups is likely to be plausible when compared with the event rate in the non-missing data.

  Two scenarios will be considered as follows:

  - Scenario A: In the usual care group, assume all missing responses are events, (i.e. adherence to guideline = "Yes") and so a value of "Yes" will be imputed. For missing responses in the intervention group, we will first replace X missing responses with event (i.e. adherence to guideline = "Yes") where X is the number of guideline adherence such that the guideline adherence rate in the missing responses is equal to the guideline adherence rate in the non-missing responses in the intervention group. For example if the guideline adherence rate in the non-missing data in the intervention group is say 20% and we have 10 missing responses we will add 0.2*10 = 2 additional patients in the intervention group as being guideline adherent. This will be regarded as the base case. All other missing responses in the intervention group will be considered as "no" not guideline adherent. The primary outcome model as specified in section 9.5 will be fitted. The CI from the treatment estimate will be examined and stored. Following the base case, an additional event will be added to the intervention group and the above procedure repeated. This process will end when the number of events added to the intervention group are equal to the original number of missing responses in this group. The tipping point for the intervention group will occur when enough events have been added such that the upper/lower limit of the CI from the corresponding model differs from that of the primary ITT finding (in regards to whether or not the CI crosses the null value).

  - Scenario B: In the intervention group, assume all missing responses are events, (i.e. adherence to guideline = "Yes") and so a value of "Yes" will be imputed. For missing responses in the usual care group, we will first replace X missing responses with event (i.e. adherence to guideline = "Yes") where X is the number of guideline adherence such that the guideline adherence rate in the missing responses is equal to the guideline adherence rate in the non-missing responses in the usual care

group. For example if the guideline adherence rate in the non-missing data in the usual care group is say 20% and we have 10 missing responses we will add 0.2*10 = 2 additional patients in the usual care group as being guideline adherent. This will be regarded as the base case. All other missing responses in the usual care group will be considered as "no" not guideline adherent. The primary outcome model as specified in section 9.5 will be fitted. The CI from the treatment estimate will be examined and stored. Following the base case, an additional event will be added to the usual care group and the above procedure repeated. This process will end when the number of events added to the intervention group are equal to the original number of missing responses in this group. The tipping point for the usual care group will occur when enough events have been added such that the upper/lower limit of the CI from the corresponding model differs from that of the primary ITT finding (in regards to whether or not the CI crosses the null value).

For both scenario A and scenario B, results will be presented visually. The event rate (i.e. adherence to guideline = "Yes") in the missing data in the intervention group (scenario A) or usual care group (scenario B) will be plotted on the x-axis. The effect size (intervention vs. usual care) from the model will be on the plotted on the y-axis. The corresponding CI will be included around each effect size. If the CI in the ITT analysis contains null value, then the point where the upper CI falls below or above the null value will be highlighted (tipping point) on the plot. If the CI in the ITT analysis does not contain the null value (i.e. one is superior to the other), then the point where the CI crosses the null value will be highlighted (tipping point). The base case will also be highlighted on each plot.

- An additional sensitivity analysis will be performed using a time-window around the follow-up assessment, i.e. only include those follow-up assessments done within 6-9 months post-randomisation

- Original statistical analysis method from version 1.0

In version 1.0 of the SAP, the chosen statistical analysis model for co-primary outcome as using a mixed effects binomial regression model. A "log" link to estimate the relative risk (RR) and an "identity" link to estimate the risk difference (RD) was proposed. In this version 2.0 of the SAP, in line with current best statistical practice, we proposed to use the marginal standard approach to estimate RR and RD (see section 9.5) as this method in comparison is less prone to model converge issues as well as protects against potential model misspecification. However since we had stated the mixed effects binomial regression model in version 1.0 of the SAP, we have decided to keep this included as a sensitivity analysis rather than removing it completely.

# 10. Analysis of sub-randomisations

Not Applicable.

## 11. Health economic analysis

No health economic analysis is planned for this trial.

## 12. Statistical software

Statistical analysis will be undertaken in the following statistical software packages: SAS software, version 9.4 (or higher) and/or Stata version 17 (or higher) will be used for all analyses.

## 13. References


1. Kirchhof P, Benussi S, Kotecha D, Ahlsson A, Atar D, Casadei B, Castella M, Diener HC, Heidbuchel H, Hendriks J, Hindricks G, Manolis AS, Oldgren J, Popescu BA, Schotten U, Van Putte B, Vardas P. 2016 ESC Guidelines for the management of atrial fibrillation developed in collaboration with EACTS. Eur Heart J. 2016;37:2893-2962

2. Hindricks G, Potpara T, Dagres N, Arbelo E, Bax JJ, Blomstrom-Lundqvist C, Boriani G, Castella M, Dan GA, Dilaveris PE, Fauchier L, Filippatos G, Kalman JM, La Meir M, Lane DA, Lebeau JP, Lettino M, Lip GYH, Pinto FJ, Thomas GN, Valgimigli M, Van Gelder IC, Van Putte BP, Watkins CL. 2020 ESC Guidelines for the diagnosis and management of atrial fibrillation developed in collaboration with the European Association of Cardio-Thoracic Surgery (EACTS). Eur Heart J. 2020; 10.1093/eurheartj/ehaa612

3. P Kirchhof, et al. Management of atrial fibrillation in seven European countries after the publication of the 2010 ESC Guidelines on atrial fibrillation: primary results of the PREvention oF thromboemolic events-European Registry in Atrial Fibrillation (PREFER in AF). Europace. 2014;16:6-14.

4. GY Lip, et al. Prognosis and treatment of atrial fibrillation patients by European cardiologists: one year follow-up of the EURObservational Research Programme-Atrial Fibrillation General Registry Pilot Phase (EORP-AF Pilot registry). Eur Heart J.2014;35:3365-76.

5. D Vinereanu, et al. A multifaceted intervention to improve treatment with oral anticoagulants in atrial fibrillation (IMPACT-AF): an international, cluster-randomised trial. Lancet. 2017;390:1737-46.

6. MK Campbell, PM Fayers, JM Grimshaw. Determinants of the intracluster correlation coefficient in cluster randomized trials: the case of implementation research. Clin Trials. 2005;2:99-107.

7. RB Schnabel, et al. Gender differences in clinical presentation and 1-year outcomes in atrial fibrillation. Heart. 2017;103:1024-30.

8. DAMOCLES Study Group. A proposed charter for clinical trial data monitoring committees: helping them to do their job well. Lancet. 2005;365:711-22.

9. Gupta SK. Intention-to-treat concept: A review. Perspect Clin Res. 2011;2(3):109-112.

10. Schulz KF, Altman DG, Moher D, for the CONSORT Group. CONSORT 2010 Statement: updated guidelines for reporting parallel group randomised trials. BMJ. 2010;340:c332.

11. Altman DG, Dore CJ. Randomisation and baseline comparisons in clinical trials. Lancet. 1990;335:149–53.

12. White IR, Horton NJ, Carpenter J, Pocock SJ. Strategy for intention to treat analysis in randomised trials with missing outcome data. BMJ. 2011;342:d40.

13. Gallis, J.A. and E.L. Turner, Relative measures of association for binary outcomes: challenges and recommendations for the global health researcher. Annals of Global Health, 2019. 85(1).

14. Kirkwood, B.R., et al., Effect of the Newhints home-visits intervention on neonatal mortality rate and care practices in Ghana: a cluster randomised controlled trial. The Lancet, 2013. 381(9884): p. 2184-2192.

15. StataCorp, Stata 17 Base Reference Manual: Margins. 2021: College Station, TX: Stata Press.

16. Wand R, Lagakos SW, Ware JH, Hunter DJ, Drazen JM. Reporting of subgroups analyses in clinical trials. NEJM. 2007;357:2189-94.

# Appendix A: Deviations from SAP

This report below follows the statistical analysis plan dated *<insert effective date of latest SAP>* apart from following:

| Section of report not following SAP | Reason |
|---|---|
| <insert section > | <insert, e.g. exploratory analyses request by TMG> |

# Appendix B: Trial schema

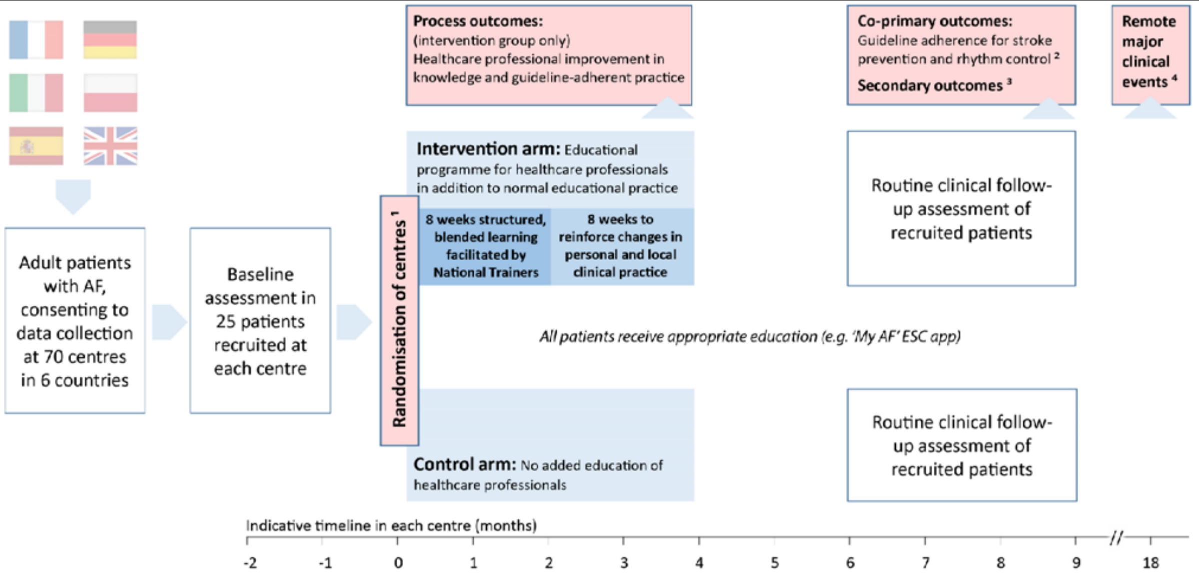


AF = atrial fibrillation; ESC = European Society of Cardiology.
[1] Minimisation algorithm accounting for country and guideline adherence for stroke prevention and rhythm control at baseline.
[2] Class I and III recommendations on stroke prevention and rhythm control therapy (ESC Guidelines on the Management of AF).
[3] Proportion of relevant guidelines attained; Appropriate use of anticoagulation; Integrated AF care approach; Patient quality of life.
[4] Composite of mortality, stroke, transient ischaemic attack, pulmonary or systolic embolus, acute coronary syndrome/myocardial infarction, heart failure and major or clinically-relevant bleeding; plus hospital admissions.

## Appendix C: Schedule of assessments

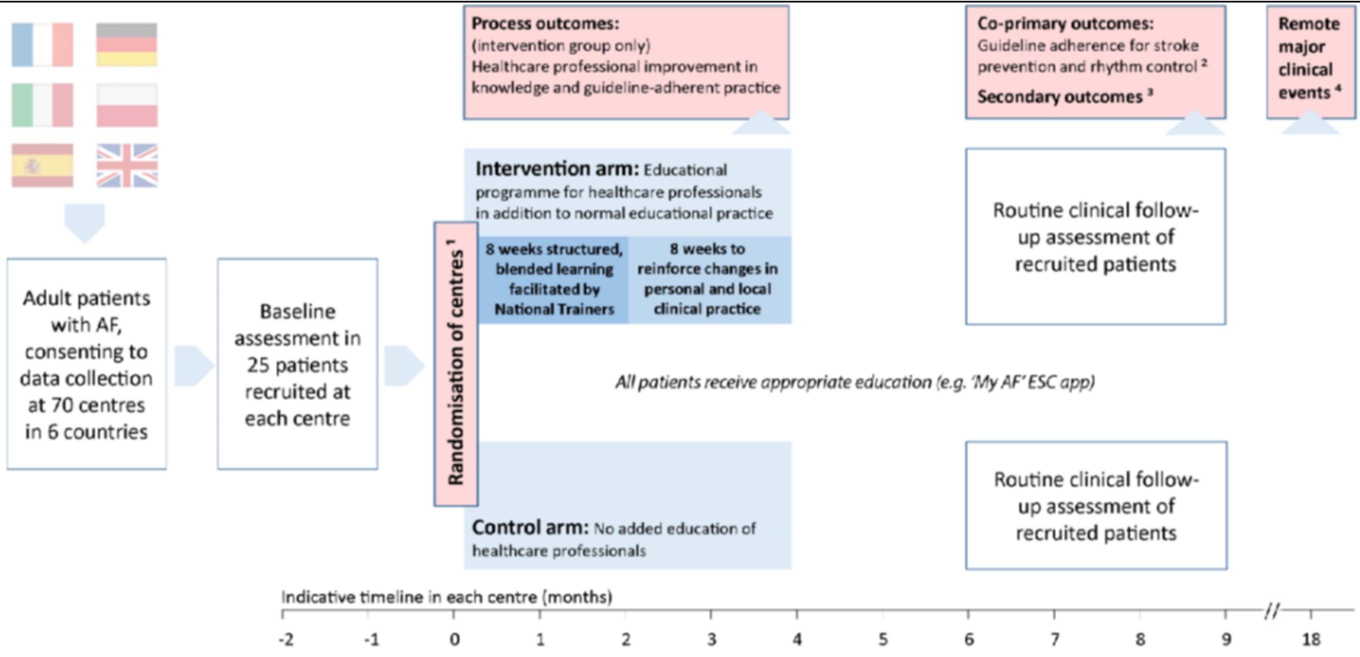


STEEER-AF

AF = atrial fibrillation; ESC = European Society of Cardiology.
[1] Minimisation algorithm accounting for country and guideline adherence for stroke prevention and rhythm control at baseline.
[2] Class I and III recommendations on stroke prevention and rhythm control therapy (ESC Guidelines on the Management of AF).
[3] Proportion of relevant guidelines attained; Appropriate use of anticoagulation; Integrated AF care approach; Patient quality of life.
[4] Composite of mortality, stroke, transient ischaemic attack, pulmonary or systolic embolus, acute coronary syndrome/myocardial infarction, heart failure and major or clinically-relevant bleeding; plus hospital admissions.

## Appendix D: Template report

A template report for the final analyses will be provided in a separate document.
