# Supplementary material for: Education of healthcare professionals to improve guideline adherence in atrial fibrillation: the STEEER-AF cluster-randomized clinical trial
Source: Nat Med. 2025 Jun 13;31(8):2647–54. doi: 10.1038/s41591-025-03751-2 (PMC12353803; doi:10.1038/s41591-025-03751-2)
Supplement: Supplementary file 2 — Reporting Summary [file 41591_2025_3751_MOESM2_ESM.pdf]

nature portfolio

Corresponding author(s): Dipak Kotecha

Last updated by author(s): Mar 31, 2025

Reporting Summary

Nature Portfolio wishes to improve the reproducibility of the work that we publish. This form provides structure for consistency and transparency in reporting. For further information on Nature Portfolio policies, see our Editorial Policies and the Editorial Policy Checklist.

Statistics

For all statistical analyses, confirm that the following items are present in the figure legend, table legend, main text, or Methods section.

n/a	Confirmed	
☐	☒	The exact sample size (*n*) for each experimental group/condition, given as a discrete number and unit of measurement
☐	☒	A statement on whether measurements were taken from distinct samples or whether the same sample was measured repeatedly
☐	☒	The statistical test(s) used AND whether they are one- or two-sided<br>*Only common tests should be described solely by name; describe more complex techniques in the Methods section.*
☐	☒	A description of all covariates tested
☐	☒	A description of any assumptions or corrections, such as tests of normality and adjustment for multiple comparisons
☐	☒	A full description of the statistical parameters including central tendency (e.g. means) or other basic estimates (e.g. regression coefficient) AND variation (e.g. standard deviation) or associated estimates of uncertainty (e.g. confidence intervals)
☐	☒	For null hypothesis testing, the test statistic (e.g. F, t, r) with confidence intervals, effect sizes, degrees of freedom and P value noted<br>*Give P values as exact values whenever suitable.*
☒	☐	For Bayesian analysis, information on the choice of priors and Markov chain Monte Carlo settings
☒	☐	For hierarchical and complex designs, identification of the appropriate level for tests and full reporting of outcomes
☒	☐	Estimates of effect sizes (e.g. Cohen's *d*, Pearson's *r*), indicating how they were calculated

Our web collection on statistics for biologists contains articles on many of the points above.

Software and code

Policy information about availability of computer code

Data collection	Participant data were collected using electronic case report forms (eCRF), managed by an independent contract research organization (Soladis; France). To provide objective assessment of the clinical care received, the eCRF was completed by the PI who was not involved in the care pathway for recruited participants. Data from investigators using the online platform were extracted by staff at the European Society of Cardiology.
Data analysis	Algorithms were used to determine guideline adherence at the level of each patient, which were finalized and approved prior to the first randomization and have been published:https://doi.org/10.1093/europace/euae178.<br>Statistical analyses for the co-primary outcomes were double-coded by an independent statistician in a separate statistical package (Stata; StataCorp, Texas) to the analyses conducted by the senior statistician (SAS; SAS Institute, North Carolina).

For manuscripts utilizing custom algorithms or software that are central to the research but not yet described in published literature, software must be made available to editors and reviewers. We strongly encourage code deposition in a community repository (e.g. GitHub). See the Nature Portfolio guidelines for submitting code & software for further information.

Data

Policy information about availability of data

All manuscripts must include a data availability statement. This statement should provide the following information, where applicable:

- Accession codes, unique identifiers, or web links for publicly available datasets
- A description of any restrictions on data availability
- For clinical datasets or third party data, please ensure that the statement adheres to our policy

Anonymized summary data will be made available for non-commercial purposes on request to the corresponding author, after completion and publication of clinical follow-up and secondary manuscripts (Prof Dipak Kotecha; d.kotecha@bham.ac.uk; 90-days response time for decisions following review by the STEEER-AF Trial Management Group).

Research involving human participants, their data, or biological material

Policy information about studies with human participants or human data. See also policy information about sex, gender (identity/presentation), and sexual orientation and race, ethnicity and racism.

Reporting on sex and gender	Gender used as this was self-identified by participants on enrollment.<br>Gender reported in main results and table 1.<br>Prespecified subgroup analyses for the co-primary outcomes included gender.
Reporting on race, ethnicity, or other socially relevant groupings	Ethnicity information on patients or healthcare staff were not collected.
Population characteristics	The mean age of participants was 68.9 years (SD 11.7), with 647 (37.4%) women.<br>The median number of beds for overnight stay per center was 700 (IQR 177 to 1180), with 40.0% general or secondary care hospitals, 25.7% tertiary care hospitals, 55.7% University hospitals and 5.7% other units.
Recruitment	70 centers across France, Germany, Italy, Poland, Spain and the United Kingdom were included, with centers recruiting a total of 1732 patients with AF, with average cluster size of 24.7 patients (coefficient of variation in cluster size 0.06). To minimize selection bias, investigators were asked to approach consecutive patients under their care that met the inclusion criteria. The requirement for participant consent means there is potential for patient self-selection bias. Investigators were a broad range of multidisciplinary healthcare professionals at that site in order to further mitigate such biases.
Ethics oversight	The trial was approved by ethical review committees and research governance authorities:<br>France: Comité de Protection des Personnes Est-II Siège : CHRU – Hôpital Saint Jacques Réf SIRIPH : 20.09.30.40105.<br>Germany: Hamburg Medical Chamber 2021-200011-BO-bet; Universität Zu Lübeck 20-403; Universität Leipzig 274/21-1k; Medizinische Hochschule Hannover Nr.9774_BO_K_2021; Schleswig-Holstein Medical Association EK/AH/EN 052/21 m; Sächsische Landesärztekammer EK-BR-59/21-1.<br>Italy: Comitato Etico di Area Vasta Emilia Centro 1285/2020/SPER/AOUMO SIRER ID2030; Comitato Etico Regionale - CER Umbria 4052/19; Comitato Etico di Area Vasta Emilia Centro della Regione Emilia-Romagna (CE-AVEC) Ethics 448/2021/Sper/AOUFe; Comitato Etico Regionale delle Marche n. cerm 2021 393; Comitato Etico Universita Federico II 186/21.<br>Poland: Heart Rhythm Disorders Clinic, National Institute of Cardiology.<br>Spain: el Comité de Ética de Investigación con Medicamentos del Hospital Universitario Vall d'Hebron PR(AG)613/2020; el Comité de ética de Investigación con medicamentos y comision dCEIM GIRONA PR(AG)613/2020.<br>United Kingdom: NHS Health Research Authority 21/PR/0040.

Note that full information on the approval of the study protocol must also be provided in the manuscript.

Field-specific reporting

Please select the one below that is the best fit for your research. If you are not sure, read the appropriate sections before making your selection.

☒ Life sciences ☐ Behavioural & social sciences ☐ Ecological, evolutionary & environmental sciences

For a reference copy of the document with all sections, see nature.com/documents/nr-reporting-summary-flat.pdf

Life sciences study design

All studies must disclose on these points even when the disclosure is negative.

Sample size	Sample size calculations for the stroke prevention co-primary outcome assumed 80% of control patients expected to receive guideline-adherent care, based on available observational studies. A relative increase of 10% was considered clinically-relevant (absolute increase from 80% to 88%). Power was 85% for this co-primary outcome, based on an intracluster correlation coefficient of 0.04, two-sided alpha 0.05, cluster size of 25 patients, coefficient of variation in cluster size 0.20, 70 clusters, and 10% loss to patient follow-up. For the rhythm control co-primary outcome, estimates of guideline-adherent care for rhythm control in the control group were 50%. Power was 85% to detect an

absolute increase from 50% to 61% using the same assumptions as the stroke prevention co-primary outcome.

Data exclusions

Intervention group: 15 patients withdrew consent, 45 lost to follow-up, 39 died prior to follow-up, 1 partial completion of follow-up.
Control group: 13 patients withdrew consent, 47 lost to follow-up, 34 died prior to follow-up, 0 partial completion of follow-up.
No cluster drop-outs.

Replication

All statistical analyses for the co-primary outcomes were double-coded by an independent statistician in a separate statistical package (Stata; StataCorp, Texas) to the analyses conducted by the senior statistician (SAS; SAS Institute, North Carolina). In addition, exploratory analyses indicated consistent effects for the co-primary outcomes across subgroups. The sensitivity analysis confirmed robust findings for both of the co-primary outcomes using a tipping point approach (presented in extended data figure 1).

Randomization

Centers were randomized only after they had finished participant recruitment and fully completed baseline electronic case report forms (eCRF), managed by an independent contract research organization (Soladis; France). To provide objective assessment of the clinical care received, the eCRF was completed by the PI who was not involved in the care pathway for recruited participants. The eCRF was completed after the interaction between patient and investigator using all available clinical and/or electronic documentation. Algorithms were used to determine guideline adherence at the level of each patient, which were finalized and approved prior to the first randomization and have been published. Randomization was performed by the Birmingham Clinical Trials Unit (University of Birmingham), with a 1:1 ratio to intervention or control using a minimization algorithm to ensure balance by (1) country; (2) cluster-specific mean for class I and III guideline adherence to stroke prevention at baseline (<70 and ≥70%); and (3) cluster-specific mean for class I and III guideline adherence to rhythm control at baseline (<50 and ≥50%).

Blinding

Guideline adherence at patient-level using the algorithms was not disclosed to the PI or investigators to avoid influencing follow-up. The randomized allocation was performed by the trial statistician blinded to the identity of the centers. Due to the nature of the intervention, it was not possible to blind investigators to the randomized allocation. The Trial Steering Committee were blinded to the randomized allocation of centers during the entire trial.

Reporting for specific materials, systems and methods

We require information from authors about some types of materials, experimental systems and methods used in many studies. Here, indicate whether each material, system or method listed is relevant to your study. If you are not sure if a list item applies to your research, read the appropriate section before selecting a response.

Materials & experimental systems

n/a	Involved in the study
☒	☐ Antibodies
☒	☐ Eukaryotic cell lines
☒	☐ Palaeontology and archaeology
☒	☐ Animals and other organisms
☐	☒ Clinical data
☒	☐ Dual use research of concern
☒	☐ Plants

Methods

n/a	Involved in the study
☒	☐ ChIP-seq
☒	☐ Flow cytometry
☒	☐ MRI-based neuroimaging

Clinical data

Policy information about clinical studies
All manuscripts should comply with the ICMJE guidelines for publication of clinical research and a completed CONSORT checklist must be included with all submissions.

Clinical trial registration

clinicaltrials.gov (NCT04396418)

Study protocol

attached as a supplemental file

Data collection

Data were collected from 70 centers across France, Germany, Italy, Poland, Spain and the United Kingdom. These centers included general or secondary care hospitals [28 (40.0%)], tertiary care hospitals [18 (25.7%)], University hospitals [39 (55.7%)] and other non-hospital sites [4 (5.7%)]. Data were collected on in-patients and out-outpatients that met selection criteria using a standardized electronic case report form. Randomization took place between May 2022 and February 2023 after completion of patient recruitment, with follow-up at 6-9 months post-randomization in the same 70 centers by the same investigators, and using the same standardized electronic case report form.

Outcomes

The co-primary outcomes were guideline-adherence for stroke prevention and rhythm control, based on class I and III ESC recommendations from the 2016 and 2020 guidelines on the management of AF. Prespecified secondary outcomes were the proportion of guidelines with adherence for stroke prevention and rhythm control, and the proportion of participants receiving anticoagulation according to class I and class I/IIa indications. The key patient-reported outcome was a score evaluating 8 domains of integrated AF management, completed by the patient after their consultation with the investigator. Patient-reported quality of life was determined using the EuroQol EQ-5D-5L questionnaire (index values and visual analogue scale). Process outcomes in the intervention group addressed the fidelity of the educational program.

Plants

Seed stocks	n/a
Novel plant genotypes	n/a
Authentication	n/a
